# Cholesterol and oxysterols in retinal neuron-glia interactions: relevance for glaucoma

**DOI:** 10.3389/fopht.2023.1303649

**Published:** 2024-01-03

**Authors:** Elodie A.Y. Masson, Jeanne Serrano, Elise Leger-Charnay, Niyazi Acar

**Affiliations:** ^1^ Eye and Nutrition Research Group, Centre des Sciences du Goût et de l'Alimentation, CNRS, INRAE, Institut Agro, Université de Bourgogne, Dijon, France; ^2^ Sensory Perception, Glia/Neuron Interaction Research Group, Centre des Sciences du Goût et de l'Alimentation, CNRS, INRAE, Institut Agro, Université de Bourgogne, Dijon, France

**Keywords:** retina, Müller cells, POAG, sterol, 24S-hydroxycholesterol, CYP46A1

## Abstract

Cholesterol is an essential component of cellular membranes, crucial for maintaining their structural and functional integrity. It is especially important for nervous tissues, including the retina, which rely on high amounts of plasma membranes for the transmission of the nervous signal. While cholesterol is by far the most abundant sterol, the retina also contains cholesterol precursors and metabolites, especially oxysterols, which are bioactive molecules. Cholesterol lack or excess is deleterious and some oxysterols are known for their effect on neuron survival. Cholesterol homeostasis must therefore be maintained. Retinal glial cells, especially Müller cells, the principal glial cells of the vertebrate retina, provide mechanical, nutritional, and metabolic support for the neighboring neurons. Several pieces of evidence indicate that Müller cells are major actors of cholesterol homeostasis in the retina, as it is known for other glial cells in the brain. This process is based on a close cooperation with neurons, and sterols can be signaling molecules participating in glia-neuron interactions. While some implication of cholesterol in age-related macular degeneration is now recognized, based on epidemiological and laboratory data, evidence for its role in glaucoma is still scarce. The association between cholesterolemia and glaucoma is controversial, but experimental data suggest that sterols could take part in the pathological processes. It has been demonstrated that Müller glial cells are implicated in the development of glaucoma through an ambivalent reactive retinal gliosis process. The early steps contribute to maintaining retinal homeostasis and favor the survival of ganglion cells, which are targeted during glaucoma. If gliosis persists, dysregulation of the neuroprotective functions, cytotoxic effects of gliotic Müller cells and disruption of glia-neuron interactions lead to an acceleration of ganglion cell death. Sterols could play a role in the glial cell response to glaucomatous injury. This represents an understudied but attractive topic to better understand glaucoma and conceive novel preventive or curative strategies. The present review describes the current knowledge on i) sterol metabolism in retinal glial cells, ii) the potential role of cholesterol in glaucoma, and iii) the possible relationships between cholesterol and oxysterols, glial cells and glaucoma. Focus is put on glia-neuron interactions.

## 1 Introduction: background information

### 1.1 Cholesterol and oxysterols

**Figure 1:**
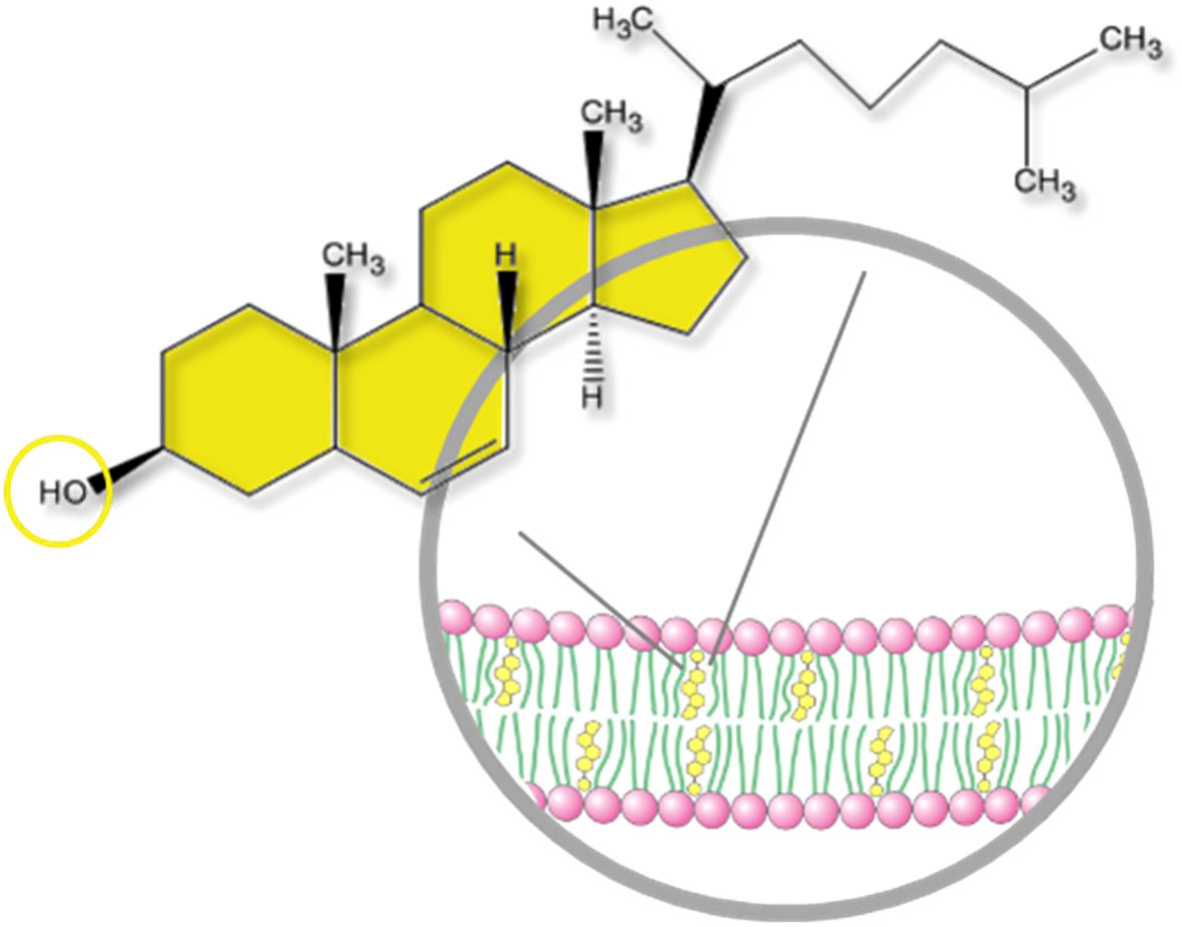
Structure of a cholesterol molecule and localization into the plasma membrane. Cholesterol is a slightly amphiphatic molecule. It is composed of a hydrophobic, non-polar body (sterane core with a carbon side chain) and a hydrophilic polar head (OH hydroxyl group). The hydroxyl group can be esterified by a fatty acid making a cholesteryl ester. Free cholesterol is integrated into the phospholipid bilayer of cellular membranes.

Circulating cholesterol is the most famous form of cholesterol because of its well-known implication in atherosclerosis and cardiovascular diseases. Cholesterol is transported in lipoproteins (LPs), which are supramolecular assemblies made of lipids (cholesterol, phospholipids and triglycerides) and apolipoproteins. Several types of LPs exist depending on their composition, size and density. The major ones are Low Density LPs (LDL) carrying cholesterol and lipids from the liver to the peripheral tissues, which uptake them to meet their needs, and High Density LPs (HDL) carrying them back to the liver, which recycles or eliminates excess. However, the great majority of cholesterol is distributed in organs, mainly in the liver, steroidogenic and nervous tissues. The brain is by far the organ with the highest cholesterol content, mainly because cholesterol is a major component of the myelin sheath, which enwraps axons. Cholesterol is also a major component of plasma membranes and therefore particularly important in nervous tissues, such as the retina, which contain high amounts of membranes (1). Cholesterol is present in all retinal layers mostly in its free (unesterified) form (2). Cholesteryl esters, which correspond to cholesterol linked to a fatty acid, a storage and transport form of cholesterol, are mostly restricted to the retinal pigment epithelium (RPE)/choroid area. Cholesterol is composed of a hydrophobic non-polar body and a hydrophilic polar head made of a hydroxyl group (**Figure 1**). This peculiar amphiphatic structure enables cholesterol to integrate into the phospholipid bilayer that constitutes cellular membranes. One of the most important role of cholesterol is to modulate the physico-chemical properties of cell membranes by interacting with phospholipids and sphingolipids (3). Cholesterol increases the degree of condensation and decreases the fluidity of cell membranes thus modulating the function of resident proteins and regulating signal transduction, membrane trafficking and ligand binding. It is of particular importance in neurons, where the biophysical characteristics of the plasma membrane determines the transmission of the nervous signal. Cholesterol was shown to modulate several aspects of neurotransmission including synapse biogenesis and function (4). In the retina, the lipid environment of the photopigments within the photoreceptor (PR) outer segments is crucial for their conformational movements at the origin of visual transduction. Cholesterol stabilizes rhodopsin while DHA (docosahexaenoic acid), the major polyunsaturated fatty acid of the retina, facilitates the protein conformational change and thus phototransduction (5). In addition to modulating membrane fluidity, cholesterol also contributes to the assembly and stabilization of lipid rafts. These are signaling platforms enriched in cholesterol, sphingolipids and signaling membrane proteins. By favoring interactions between specific partners, rafts are thought to be implicated in the activation of numerous signaling cascades (6).

**Figure 2:**
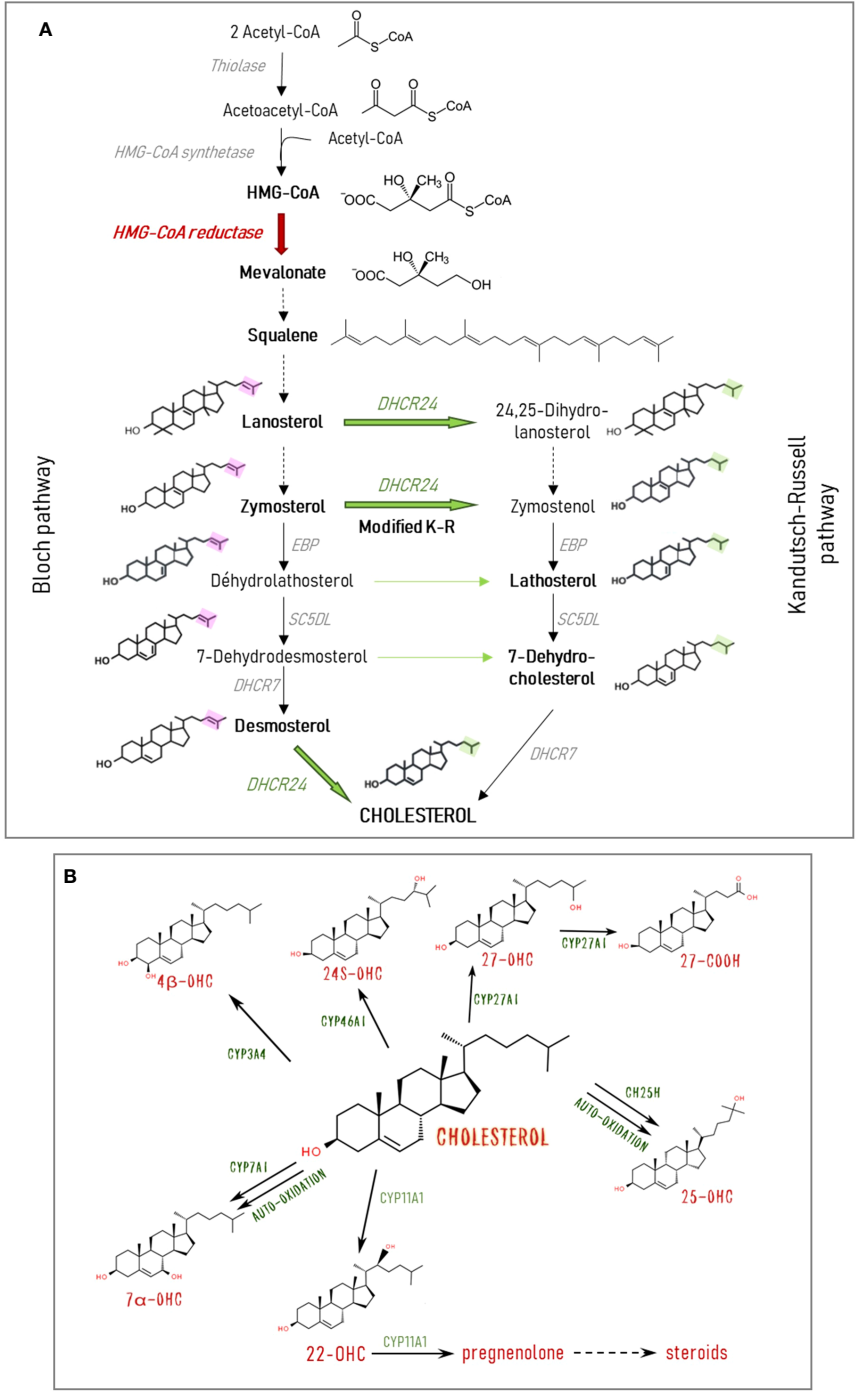
Cholesterol and oxysterol biosynthesis pathways. **(A)** Cholesterol is synthesized from acetyl-CoA through a multi-enzymatic process taking place mainly in the endoplasmic reticulum. The rate-limiting enzyme HMG-CoA (3-hydroxy-3-methylglutaryl-CoA) reductase produces the key intermediate mevalonate. The two major parallel pathways of cholesterol biosynthesis are represented: the Kandutsch-Russell (K-R) and the Bloch pathways. They differ in the step of the side chain reduction by the DHCR24 (24-dehydrocholesterol reductase) and the consecutive precursors involved. It is thought that cholesterol is primarily synthesized through the Bloch pathway in astrocytes, while neurons use the K-R pathway. Cross-over sites between the two pathways exist via the action of DHCR24. In the modified K-R pathway proposed by Mitsche et al. (7) the predominant action of this enzyme is to convert zymosterol from the Bloch pathway into zymostenol in the K-R pathway. Solid arrows represent one reaction. Broken arrows represent several reactions, and only initial substrate and final product are shown. **(B)** Cholesterol can undergo both enzymatic and non-enzymatic oxidation. The major oxysterols found in the retina and the implicated enzymes are shown. CH25H, cholesterol 25-hydroxylase; CYP3A4, cytochrome P450 family 3 subfamily A member 4 (cholesterol 4β-hydroxylase); CYP7A1, cytochrome P450 family 7 subfamily A member 1 (cholesterol 7α-hydroxylase); CYP11A1, cytochrome P450 family 11 subfamily A member 1 (cholesterol 22-hydroxylase); CYP27A1, cytochrome P450 family 27 subfamily A member 1 (cholesterol 27-hydroxylase); CYP46A1, cytochrome P450 family 46 subfamily A member 1 (cholesterol 24-hydroxylase); DHCR7, 7-dehydrocholesterol reductase; DHCR24, 24-dehydrocholesterol reductase; EBP, 3-beta-hydroxysteroïd delta; SCD5L, lathosterol oxidase.

While cholesterol itself is by far the most abundant sterol in the retina, as in other mammalian tissues, its precursors and metabolites, especially the bioactive oxysterols, are also present. Cholesterol biosynthesis implicates a series of reactions generating many precursor molecules (**Figure 2A**). Cholesterol and precursors can undergo both enzymatic and non-enzymatic oxidation [for a review see (8)]. The main oxysterols present in the retina are shown in **Figure 2B**. Oxysterols are elimination products of cholesterol since they acquired the ability to cross membranes and barriers freely contrary to cholesterol itself. However, data are accumulating showing that they are also signaling molecules playing multiple roles. They are precursors of steroid hormones, including neurosteroïds such as pregnenolone. 24S-hydroxycholesterol (24S-OHC), the major oxysterol of the brain also found in the retina, has been shown to affect neuron viability *in vitro* (9–13) and to modulate neuronal signaling and synaptic plasticity (11, 14). Oxysterols were also found to be the ligands of Liver X receptors (LXRs), nuclear transcription factors regulating target genes implicated in cholesterol metabolism (8).

Cholesterol homeostasis needs to be maintained in order to ensure structural and functional integrity of cells and tissues. There are several examples of neurological disorders associated with dysregulation of cholesterol metabolism showing that deficiency or cholesterol accumulation is deleterious for the nervous system (15). This is the case of the inherited metabolic diseases Smith-Lemli-Opitz syndrome (SLOS) and Niemann-Pick type C (NPC). Interestingly, retinal alterations have been documented in patients (16) and in rodent models of the diseases (17, 18). Cholesterol also seems to be implicated in common neurodegenerative diseases of the central nervous system (CNS) such as Alzheimer, Parkinson and Huntington (15, 19, 20). While the link between plasma cholesterol levels and these pathologies remains unclear and controversial, increased free cholesterol levels have been measured in brain regions of Alzheimer’s disease (AD) patients (21, 22). Moreover, experimental data obtained from manipulation of cholesterol metabolism in rodents also support a role of cholesterol in neurodegenerative diseases (23–25). Regarding retina-specific diseases, cholesterol has been shown to accumulate in the aging Bruch’s membrane, at the base of the retina, during age-related macular degeneration (AMD). It is a major component of the lipid-rich deposits (drusen) typical of the disease (26). It is noticeable that many variants that have been associated with AMD in GWAS (Genome Wide Association Study) studies are located in genes involved in cholesterol metabolism (27).

### 1.2 Glial cells and their interactions with neurons

As a nervous tissue, the retina encompasses neurons but also glial cells, namely microglial cells, astrocytes and Müller cells. Neurons are specialized in generating and exchanging electrical signals. Microglial cells are the blood-derived resident immune cells. Astrocytes and Müller cells are macroglial cells providing structural, nutritional and metabolic support for neurons. Müller cells are the most predominant macroglial cells and will be the focus of this review even though astrocytes and microglial cells perform important and specific functions in the retina as well. Müller cells extend almost the entire thickness of the retina. Their radial and bipolar structural organization enables them to connect different cell types and retinal compartments. It is considered that a Müller cell constitutes a functional unit with neighboring neurons, and meets their requirements by endorsing plenty of functions [for a review, see (28, 29)]. They regulate extracellular space volume, ion and water homeostasis. They provide nutrients to neurons (glucose, lactate, *etc.*), and remove metabolic waste. They participate in the inner blood-retina barrier (BRB) and in the regulation of retinal blood flow. They uptake and recycle excess glutamate, the main excitatory neurotransmitter in the retina, providing neurons with neurotransmitter precursors. Müller cells also release gliotransmitters (glutamate, ATP, adenosine) and thus participate in synaptic activity. They synthesize neurotrophic factors and cytokines required for neuron survival such as BDNF (Brain-derived neurotrophic factor), CNTF (Brain ciliary neurotrophic factor) or FGF (Fibroblast growth factor), as well as anti-oxidants such as glutathione. Müller cells also participate in lipid metabolism by incorporating the major retinal polyunsaturated fatty acid, DHA, into phospholipids and channeling it to photoreceptors. Evidence that Müller cells contribute to retinal cholesterol metabolism and homeostasis will be detailed in this review.

It is well-admitted that glia-neuron interactions participate in retinal homeostasis and maintain a proper environment required for the intense neuronal activity. Interestingly, the nature of these interactions is thought to be different under normal, early and late pathological conditions. Indeed, retinal macroglial cells, astrocytes and Müller cells, are known to become activated in response to various stresses and pathological alterations. This process, called reactive gliosis, include morphological, biochemical and physiological changes considered as an attempt to protect the retina from further damage and to preserve nervous function. The neuroprotective effects consist in the release of neurotrophic factors and antioxidants, glutamate uptake and facilitation of neovascularization. Müller cells might even transdifferentiate to neuronal progenitors to regenerate lost neurons. However, at later stages and/or in more severe cases of gliosis, the supportive functions of Müller cells are impaired thus increasing the susceptibility of neurons to stressful stimuli in a diseased retina (30, 31). Dedifferentiation of Müller cells is associated with functional uncoupling from neurons. Retinal homeostasis is no longer maintained and glutamate toxicity occurs resulting in neuron degeneration. Moreover, gliotic Müller cells release pro-inflammatory cytokines and produce excess nitric oxide (NO). Their hypertrophy, proliferation and migration cause a glial scar, which impedes neuronal regeneration and results in in aberrant tissue repair.

### 1.3 Glaucoma

Glaucoma is a retinal neurodegenerative disease and the leading cause of irreversible blindness worldwide. It encompasses a heterogeneous group of optic neuropathies characterized by a specific deformation of the optic nerve head and the progressive degeneration of retinal ganglion cells (RGCs) and their axons, which constitute the optic nerve [for a review, see (32)]. Glaucoma is considered to be a multifactorial disease. Older age and elevated intraocular pressure (IOP) are the major risk factors for primary open-angle glaucoma (POAG), the most common form of glaucoma. The principal mechanism proposed to explain the pathogenesis of glaucoma is based on the idea that elevated IOP, due to an excess of aqueous humor in the anterior part of the eye, exerts a mechanical compression on the optic nerve head that directly damages RGCs and their axons. Until now, there is no curative treatment and lowering IOP by topical drugs, laser therapy or surgical intervention, is the current approach to slow down the progression of the disease. However, glaucoma can also occur under normal IOP. Several molecular mechanisms have been proposed to explain RGC apoptosis: failure of axonal transport leading to deprivation of neurotrophic factors; ischemia leading to oxidative stress; excitotoxicity, which corresponds to cellular toxicity induced by excess glutamate; and production of pro-inflammatory cytokines by glial cells (33).

As mentioned in the previous section, Müller cells can participate in neuronal damage under conditions of retinal stress through reactive gliosis. Glial cells (Müller, astrocytes and microglial) activation and dysfunction are thought to contribute to the pathogenesis of glaucoma (34). Müller cell gliosis has been reported in a context of glaucoma in both humans (35) and rodents (36–39). The impairment of Müller cell functions that typically support RGCs will favor their death. For example, decreased glutamine synthetase required for glutamate recycling, increased production of TNFα (tumor necrosis factor α) and NO have been shown in glaucoma models (33). A role for ATP release has also been proposed (40). The sensitivity of Müller cells to RGC damage and their reactivity to respond to it prompt to work for a better understanding of the specific role of Müller cells in glaucoma pathogenesis. Deciphering the signaling mechanisms is crucial for the development of novel preventive and therapeutic strategies.

The observations supporting an implication of cholesterol in the pathogenesis of glaucoma are not as abundant as for AMD and will be developed in this review (sections 3 and 4). Evidence for the role of cholesterol in the pathogenesis of other neurodegenerative diseases of the CNS, as mentioned above, reinforces the relevance of studying cholesterol metabolism in the context of glaucoma.

The present review aims to give an overview of cholesterol metabolism in the retina focusing on the role of Müller glial cells, in physiologic conditions as well as in glaucoma. Special interest is put on glia-neuron interactions. The review relies on data obtained from animal studies or *in vitro* studies on animal-derived retinal cells. It also includes epidemiological and clinical data in humans to document the potential role of sterols in glaucoma.

## 2 Cholesterol metabolism in retinal glial cells and neuron-glia interactions

As part of the CNS, the retina shares many features with the brain. Cholesterol metabolism as well as neuron-glia interactions and cooperation have been more widely studied in this organ. This is the reason why some relevant information relative to the brain is provided in the present section.

### 2.1 General considerations about cholesterol metabolism in the retina

Cholesterol in the body originates both from the diet and endogenous biosynthesis. Systemic cholesterol homoeostasis is ensured by an efficient LP shuttle between the intestine, which takes up dietary cholesterol, the liver, which is the main site of cholesterol biosynthesis and excretion, and peripheral organs *via* the blood circulation. The retina exhibits specificities that have direct consequences on its cholesterol metabolism. First, the continuous renewal of the PR outer segments to enable an efficient phototransduction process implies an important turnover of lipids that are present in their membranes. This is primarily taken care of by the RPE cells. Second, the BRB is permeable to cholesterol and LPs, contrary to the blood-brain barrier (BBB), which isolates the brain from the systemic LP circuit (1). Consequently, retinal cholesterol input relies both on local *de novo* biosynthesis and circulating LPs conveying cholesterol from dietary and hepatic origin. Cholesterol efflux can also rely on the LP system, in addition to a specific local pathway, namely oxysterol production. A series of reviews have been published focusing on cholesterol metabolism in the retina by Fliesler and Bretillon (41); Pikuleva and Curcio (26) and more recently by Ramachandra Rao and Fliesler (42).

#### 2.1.1 Uptake and biosynthesis of cholesterol

Cholesterol uptake from circulating LPs takes place mainly at the choroid– RPE interface, which constitutes the outer BRB. The capillaries of the choroid are fenestrated and are separated from the RPE by its basal membrane, the Bruch’s membrane. The RPE is made of a single layer of epithelial cells with tight junctions, which are in charge of selective transport of various substances including lipids. *In vivo* experiments performed in rats using labeled LDL and HDL particles with a fluorescent cholesterol analogue, injected intravenously, showed that the retina is capable of rapid uptake of circulating LPs. This process occurred in the RPE, preferentially with LDL, and fluorescence was then detected up to the PR outer segments indicating that the uptake of LPs by the RPE allows cholesterol delivery to the outer neural retina (43). It has been shown in mouse, monkey and human retinas that RPE cells express various LP receptors at their basal membrane facing the choriocapillaries: LDLR (low density lipoprotein receptor), SRB-I (scavenger receptor class B type I), SRB-II and CD36 (cluster of differentiation 36) (43–46), which enable LP uptake. Contrary to the outer BRB, the inner BRB has a similar structure to the BBB indicating that the uptake of LPs at this site is likely very minimal.

Evidence for a substantial retinal cholesterol biosynthesis was provided *in vivo* many years ago by Fliesler et al. using intravitreous injection of a deuterated substrate in the rat. The authors showed that the radiolabeled precursor was efficiently incorporated into retinal cholesterol (47, 48). More recently, Lin et al. estimated that retinal biosynthesis represents more than 70% of the cholesterol input in the mouse retina (49). Endogenous biosynthesis might therefore be the main source of cholesterol in the retina even if the estimation of retinal cholesterol biosynthesis rate and turnover varies depending on species and studies. Supporting retinal *in situ* cholesterol biosynthesis, gene and protein expression of many enzymes of the cholesterol biosynthesis pathway have been measured in the retina, including HMGCR (3-hydroxy-3-methylglutaryl Coenzyme-A reductase), the rate limiting enzyme of the cholesterol biosynthesis pathway (46, 50).

#### 2.1.2 Intraretinal cholesterol transport

Within the brain, cholesterol is transported between cells via specific LPs called HDL-like (51). They have similar size and density to plasma HDL but specific apolipoproteins. Indeed, the apolipoprotein ApoE is considered to be the main lipid transporter in the CNS, even though HDL-like also contain ApoA-I and ApoJ. The mechanism responsible for the formation of nascent LPs in the CNS is not completely understood and could involve both direct secretion of lipidated ApoE and secretion of lipid-poor or even lipid-free ApoE further lipidated extracellularly. It seems that transporter ABCA1 (ATP-binding cassette transporter A1) is a major contributor to the lipidation of ApoE-containing LPs by mediating the ATP-coupled efflux of cholesterol and phospholipids from the cells. Other transporters of the family that are ABCG1 and ABCG4 are also expressed in the brain (52). The presence of these lipid transport proteins within the neural retina and RPE supports the existence of an intraretinal transport of cholesterol mediated by LPs. ABCA1 and ABCG1 were shown to be expressed in all cell layers of the mouse retina using immunofluorescence (53). At the apical membrane of RPE cells, these transporters enable the delivery of exogenous cholesterol to the neural retina using ApoA-I, ApoE (54) and ApoJ (55). The monkey and human neural retinas have been shown to express most of the major proteins involved in lipid transport such as ABCA1, ApoA-I, ApoE, SRB-I, SRB-II, CD36 using immunofluorescence. The authors suggested that intraretinal lipid transport relies, as in the brain, on HDL-like LPs assembled in the retina (43, 45, 46). LPs are uptaken by the cells thanks to specific receptors in order to meet their needs in lipids. In the brain, LDLR and LRP1 (LDLR-related protein 1) are mainly in charge. They both recognize ApoE as a ligand that is used for LP internalization (51). LDLR expression was detected in the monkey and human retinas (43, 45, 46). The presence of LRP1, a major receptor for ApoE, was not documented in these studies but has been reported in the mouse retina (56). LRP1 expression was shown to be very weak in human retinas (57) and limited in the rat retina (58). The perturbation of retinal cholesterol metabolism and levels in mouse models with ablation of apolipoprotein genes: ApoE (59) and ApoJ (55) supports the importance of these players in cholesterol transport in the retina (see **Table 1**). LPs are known to undergo some maturation *via* the action of remodeling enzymes such as LCAT (lecithin–cholesterol acyltransferase) and CETP (cholesteryl ester transfer protein). LCAT transfers a fatty acid from phospholipids to free cholesterol forming cholesteryl esters, which can then be transferred to the HDL core, and CETP ensures the exchange of lipids between LPs. The detection of LCAT and CETP expression in the retina suggests the capacity of this tissue to engage into maturation of LPs (45).

**Table 1:** Retinal phenotype of mouse models with “knock-out” of genes implicated in cholesterol metabolism.

Mouse model	Cholesterolemia	Retinal cholesterol metabolism (vs WT*)	Retinal phenotype (vs WT*)	Author (Year)
***Cyp27a1* KO**	Normal CHO ↑cholestanol ↓ bile acid pool ↑ hepatic CHO synthesis	↑ CHO in males ↑ lanosterol, lathosterol (↑ CHO synthesis) ↑ pregnenolone, 7kCHO CHO deposit in abnormal blood vessels, in RPE-BrM in retinal lesions	Retinal-choroidal anastomosis OPL disorganization Fibrosis in RPE-BrM Glial activation Hypoxia	Omarova et al. (2012) (60)
***Cyp46a1* KO**	Normal CHO	↑ CHO: eCHO in PR and RPE basal membrane ↑ 27-COOH ↑ *Abcg1*, *Lxr*	Vascular abnormalities at 6 months (venous beading, tortuosity, microaneurisms, ↑ vascular permeability) Micro/macroglial activation Normal ERG	Saadane et al. (2019) (61)
***Cyp46a1* KO**	NA	NA	↑ IOP ↑ cup to disk ratio ↓ RGC function (PERG ampl.) at 7-8 months	El-Darzi et al. (2023) (55)
***Cyp27a1*/*Cyp46a1* DKO**	Normal CHO	↑ CHO, cholestanol ↓ uCHO in males ↑ eCHO in PR outer segments CHO deposition at RPE CHO deposition and oxidation in vascular wall → lanosterol, lathosterol, desmosterol → pregnenolone ↑ *Cyp46a1 (unfunctional)*	Vascular abnormalities (retinal-choroidal anastomosis, arteriovenous shunts, ↑ vascular permeability, nonperfusion, capillary degeneration) Müller + Macrophage activation Oxidative stress in PR inner segments ↓ retinal function from 6 months (ERG ampl)	Saadane et al. (2014) (62) Saadane et al. (2016) (63)
***Cyp27a1*/*Cyp46a1*/*Acat* TKO**	NA	↓ eCHO ↑ uCHO in PR outer segments ↓ lathosterol ↑ cholestanol	Retinal degeneration with age (apoptotic PRs)	Saadane et al. (2016) (63)
**ApoB100, *Ldlr* KO**	↑ CHO	↑ eCHO at RPE basement	↓ retinal function (ERG ampl.) with age	Bretillon et al. (2008) (64)
***ApoE* KO**	↑ CHO	↑ CHO ↑ lathosterol, desmosterol ↓ 27-COOH, →24S-OHC ↑ ApoA4 ↑ *ApoB*, ↓ *Idol, Apoc3, Apod*	Normal retinal appearance Glial activation	Saadane et al. (2018) (59)
***ApoJ* KO**	NA	↓ CHO, uCHO, eCHO ↓ lathosterol, desmosterol ↓ 24S-OHC, 7HCA ↑ 27-OHC, 27-COOH	Normal fundus ↑ cup to disk ratio Modest ↑ IOP → RGC number at 6 months ↓ RGC function (PERG ampl.) from 6 months → Müller, microglia activation	El-Darzi et al. (2023) (55)
***ApoJ* KO + efavirenz**	NA	↑ 24S-OHC ↓ 27-OHC ↑ CHO, eCHO *↑ Abcg1, ApoA-I, Scarb1, Hmgcr*	↓ IOP ↑ RGC function (PERG ampl.)	
***Abca1* KO**	NA	NA	→ RGC number at 3 months ↓ RGC number at 12 months (apoptotic RGCs) Apoptosis in INL and OPL at 12 months → IOP Retinal inflammation	Shinozaki et al. (2022) (65)
***Abca1* KO**	No detectable HDL-C ↓ CHO	↑ CHO at 3-6 months	↓ RGC number with age (apoptotic RGCs) Axon demyelination Abnormal energy production in RGCs and bipolar cells More active metabolic status Altered mitochondrial function ↓ autophagy flux	Yang et al. (2023) (66)
***Abca1* KO + atorvastatin**	NA	↓ CHO in IPL and RGC layers	↑ RGC number (no apoptotic RGCs) ↑ mitochondrial function and autophagy flux	
**Macrophage-specific *Abca1/g1* KO** (*Abca1* ^flox/flox;^ *Abcg1* ^flox/flox;^ LysM-Cre)	NA	Age-associated (6-12 months) extracellular cholesterol-rich deposits underneath neurosensory retina Accumulation of oxysterols and eCHO	BrM thickening Macrophages/microglia infiltration in subretinal space RPE dysfunction (impaired dark adaptation) PR dysfunction and degeneration	Ban et al. (2018) (67)
**Rod-specific *Abca1/g1* KO** (*Abca1* ^flox/flox;^ *Abcg1* ^flox/flox;^ Rhod-Cre)	Normal HDL-C, LDL/VLDL-C and ApoA-I	↑ CHO ↑ lipid droplets in RPE (CHO + several oxysterols – no eCHO) at 12 months	Rod dysfunction (↓ ERG ampl.) at 12 months PR degeneration at 12 months Cone dysfunction at 18 months Structural disruption of RPE	Ban et al. (2018) (68)
**Cone-specific *Abca1/g1* KO** (*Abca1* ^flox/flox;^ *Abcg1* ^flox/flox;^ HRGP-Cre)	NA	NA	No PR degenerative phenotype	Ban et al. (2018) (68)
**Glia (astrocyte)-specific *Abca1* KO** (*Abca1* ^flox/flox;^GFAP-Cre)	NA	↑ CHO in NFL (astrocytes) ↓ CHO in GCL, IPL ↓ CHO in aqueous humor	→ RGC number at 3 months ↓ RGC number at 12-18 months (apoptotic RGCs) → IOP Swelling of ON and regional loss Thinning of inner retinal layers Inner retinal dysfunction (↓ mfERG) Retinal inflammation	Shinozaki et al. (2022) (65)

Abcg1, ATP-binding cassette transporter G1; Acat, acyl-coenzymeA:cholesterol acyltransferase; Apo, Apolipoprotein; BrM, Bruch’s membrane; CHO, cholesterol; Cyp27a1, cytochrome P450 family 27 subfamily A member 1; Cyp46a1, cytochrome P450 family 46 subfamily A member 1; DKO, double KO; eCHO, esterified cholesterol; ERG, electroretinogram; GCL, ganglion cell layer; HDL-C, high density lipoprotein cholesterol; Hmgcr, HMG-CoA reductase; Idol, inducible degrader of LDL receptor; INL, inner nuclear layer; IOP, intraocular pressure; IPL, inner plexiform layer; LDL-C, low density lipoprotein cholesterol; Lxr, Liver X receptor; mfERG, multifocal electroretinogram; NA, non available data; NFL, nuclear fiber layer; ONL, outer nuclear layer; OPL, outer plexiform layer; PERG, pattern electroretinogram; PR, photoreceptor; RGC, retinal ganglion cell; RPE, retinal pigment epithelium; Scarb1, scavenger receptor class B type 1; TKO, triple KO; uCHO, unesterified cholesterol; VLDL-C, very low density lipoprotein cholesterol. Upwards (↑), downwards (↓) and horizontal (→) arrows indicate increases, decreases and no change, respectively, as compared with wild-type (WT) except * for *ApoJ* KO + efavirenz vs *ApoJ* KO and *Abca1* KO + atorvastatin vs *Abca1* KO. Gene symbols are italicized.

#### 2.1.3 Efflux of cholesterol from the retina

The permeability of the BRB not only enables the uptake of cholesterol and other lipids carried by circulating LPs but also the efflux of such molecules from the retina. The expression of ABCA1 was measured at both the apical and basal sides of the RPE cells suggesting that RPE may be able of transporting LPs both into and out of the neural retina (45). Cholesterol accumulation in the retina of *Abca1* KO mice demonstrates the crucial role of this transporter in retinal cholesterol efflux (66) (**Table 1**). Moreover, atypical LPs were isolated from aged human RPE-choroid. These were distinct from typical plasma LPs in their density profile, lipid composition and size and were also characterized by the presence of ApoB-100. Based on this characterization, Li et al. suggested that the retina is indeed able to assemble and secrete LP-like particles into the circulation (69). Those particles, called Bruch’s membrane LPs, rich in cholesteryl esters, and excreted through the basal side of the RPE and to the Bruch’s membrane could then reach the systemic circulation *via* the choroid but they also tend to accumulate in the subretinal space with advancing age, thus generating a “lipid wall” linked to AMD pathogenesis (70).

The elimination of excess cholesterol from the retina also involves another pathway: its conversion into oxysterols produced by side-chain oxidation *via* the action of cholesterol hydroxylases. Oxysterols can freely cross cellular membranes. In the brain, 24S-hydroxycholesterol (24S-OHC), product of the CYP46A1 enzyme (cytochrome P450 family 46 subfamily A member 1), is the main pathway of cholesterol elimination (71). 24S-OHC diffuses through the BBB and reaches the systemic circulation to be cleared by the liver. CYP46A1 is a microsomal monooxygenase that catalyzes hydroxylation of the side-chain of cholesterol. It utilizes heme iron receiving electrons from NADPH to produce an oxyferryl intermediate responsible for the attack of the cholesterol substrate. It abstracts a hydrogen from the carbon-24 creating a cholesterol alkyl radical, which will then be oxygenated into 24S-OHC (72). CYP46A1 has also been found to further metabolize 24S-OHC into 24,25- and 24,27-dihydrxycholesetrols. In addition, it has been shown to metabolize structurally diverse steroids and marketed drugs (73). In the retina, cholesterol oxidation involves three enzymes: CYP46A1, CYP27A1 (cytochrome P450 family 27 subfamily A member 1) and CYP11A1 (cytochrome P450 family 11 subfamily A member 1). Using RT-PCR and immunohistochemistry, Bretillon et al. showed that CYP46A1 is specifically expressed in the neural retina in the rat and its product 24S-OHC was measured in bovine neural retina (74). However, unlike in the brain where up to 75% of cholesterol is eliminated in the form of 24S-OHC, the most abundant oxysterol in the human and bovine retinas is 27-COOH (3β-hydroxy-5-cholestenoic acid), the product of the CYP27A1 enzyme (75). Accordingly, gene expression of *Cyp46a1* was shown to be much lower in the retina than in the brain, contrary to *Cyp27a1* and *Cyp11a1* (46). The increased retinal cholesterol levels in *Cyp27a1* KO (60), *Cyp46a1* KO (61) and especially double KO mice (62) indicate the importance of these enzymes for cholesterol elimination from the retina. The increase was the result of an accumulation of cholesteryl esters mainly localized to the PRs and the basal membrane of the RPE, as well as in the vascular wall of abnormal retinal blood vessels. It is noticeable that the deficiency of CYP46A1 in the *Cyp46a1* KO model was compensated by an increase of cholesterol efflux *via* 27-COOH (61). Those data are presented in **Table 1**.

**Figure 3:**
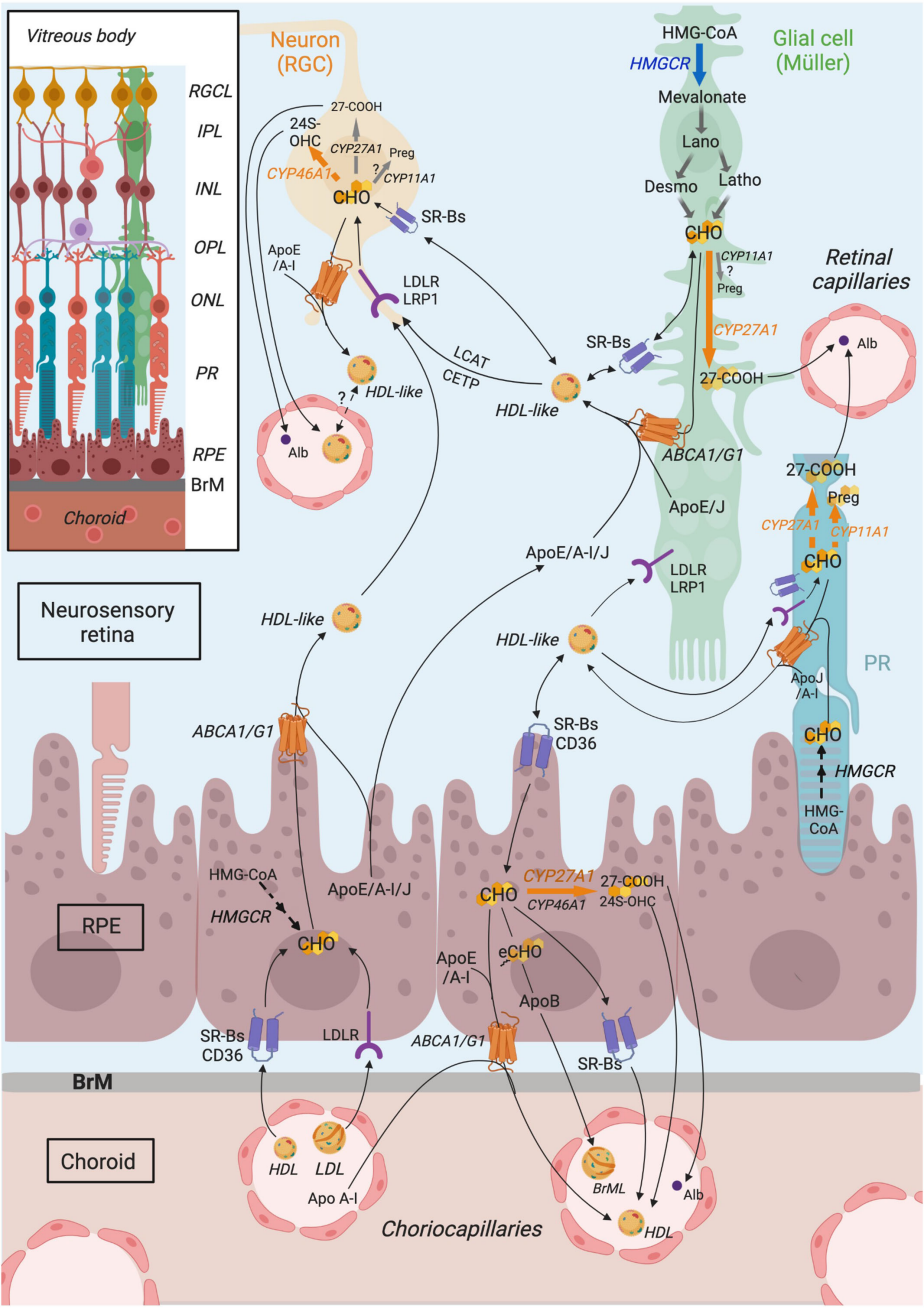
Schematic representation of retinal cholesterol metabolism proposed from compilation of published data. Cholesterol in the retina originates from a local biosynthesis, which likely occurs primarily in glial cells, which express HMGCR (HMG-CoA reductase) enzyme, even though cholesterol biosynthetic pathway might also be active in the RPE (retinal pigment epithelium) and neurons, especially photoreceptors (PRs), to a lower extent. Retinal cholesterol also comes from the uptake of blood lipoproteins by RPE cells, expressing LDLR family receptors (LDLR) and scavenger receptors (SRBs, CD36). Within the retina, cholesterol is thought to be transported in HDL (High density lipoprotein)-like particles, rich in ApoE/A-I/J, secreted by RPE and glial cells, thanks to ABC (ATP-binding cassette) transporters. Those particles mature further *via* the action of LCAT (lecithin:cholesterol acyltransferase) and CETP (cholesteryl ester transfer protein). HDL-like could be subsequently captured by neighboring cells, especially neurons, or could potentially cross the blood retina barrier to reach the systemic circulation and be eliminated. It is thought that neurons as well are able to efflux excess cholesterol via intraretinal lipoproteins. RPE cells secrete cholesterol-rich particles, including the specific Bruch’s membrane particles (BrML) at their basal membrane, which reach the circulation via the choroidal capillaries. Cholesterol output also involves its conversion into oxysterols, the main two being 24(S)-hydroxycholesterol (24S-OHC) and 3β-hydroxy-5-cholestenoic acid (27-COOH), produced by CYP46A1 and CYP27A1 enzymes, respectively. CYP46A1 is mainly expressed in RGCs (retinal ganglion cells). CYP27A1 is more ubiquitously expressed in the retina, including likely PRs and Müller cells. Pregnenolone production by CYP11A1 also occurs in the retina, in PRs and possibly in RGCs and Müller cells. Focus is put on Müller cells although other glial cell types, such as astrocytes and microglial cells, also participate in retinal cholesterol metabolism. Connections of cholesterol metabolism between cell types have been extrapolated from the literature data. ABCA1, ATP-binding cassette transporter A1; ABCG1, ATP-binding cassette transporter G1; Alb, albumin; Apo, apolipoprotein; BrM, Bruch’s membrane; CD36, cluster of differentiation 36; CETP, cholesteryl ester transfer protein; CHO, cholesterol; CYP11A1, cytochrome P450 family 11 subfamily A member 1; CYP27A1, cytochrome P450 family 27 subfamily A member 1; CYP46A1, cytochrome P450 family 46 subfamily A member 1; Desmo, Desmosterol; eCHO, Cholesteryl esters; HDL, high density lipoprotein; HMG-CoA, 3-hydroxy-3-methylglutaryl-CoA; HMGCR, HMG-CoA reductase; Latho, Lathosterol; IPL, Inner plexiform layer; INL, Inner nuclear layer; LCAT, lecithin,cholesterol acyltransferase; LDL, low density lipoprotein; LDLR, low density lipoprotein receptor; LRP1, LDLR-related protein 1; OPL, Outer plexiform layer; ONL, Outer nuclear layer; RGC, retinal ganglion cell; RGCL, RGC layer; PR, Photoreceptor; Preg, pregnenolone; RPE, retinal pigment epithelium; SRB, scavenger receptor class B; 24S-OHC, 24S-hydroxycholesterol; 27-COOH, 3β-hydroxy-5-cholestenoic acid. Adapted from (39) CC BY 4.0. Created in Biorender.com. and reprinted under a CC BY 4.0 license; with permission from Biorender; original copyright 2021.

This information regarding cholesterol metabolism in the retina has been summarized in **Figure 3**.

#### 2.1.4 Cholesterol metabolism in aging

Aging is the main risk factor for several retinal neurodegenerative diseases, including glaucoma. It is also associated with disturbances in cholesterol metabolism.

In the CNS, aging induces decreased cholesterol content in neurons and specific brain regions, such as the hippocampus. This is likely the result of both decreased cholesterol biosynthesis, especially in astrocytes, and increased neuronal *Cyp46a1* expression in response to oxidative stress (76–78). 24S-OHC levels tend to decrease in the brain and increase in the plasma during aging and in the early stages of AD (20). Aging is also associated with a redistribution of membrane cholesterol that influences membrane protein function. Demyelination is observed during aging leading to an accumulation of cholesterol-rich myelin debris, especially in microglia which exhibits reduced clearance capacity. These disturbances of cholesterol homeostasis possibly contribute to age-related cognitive decline by inducing synaptic loss and neuronal death (76). However, authors proposed that the cholesterol loss is actually required for survival under the high stress of aging by promoting the tyrosine kinase B activity and consecutive pro-survival cascade (12).

In the retina, accumulation of lipidic material within the Bruch’s membrane, under the RPE, is a typical feature of normal aging. Starting by the fourth decade of life, it is strongly age-related. Cholesteryl esters were identified as a major component of these deposits resulting in the formation of a “lipid wall” and impairing the physiology of the RPE and the transport of molecules to and out of the retina through the blood retina barrier. These lipids seem to originate from the RPE that secretes large apoB-containing lipoproteins rich in cholesteryl esters. Free cholesterol is also present and might come from photoreceptor outer segments, plasma lipoproteins and/or endogenous biosynthesis. This process is especially relevant for AMD physiopathology since the lipid-rich extracellular lesions called drusen, which are specific features of the disease, subsequently form at the location of these age-related deposits (79). Pro-inflammatory and cytotoxic compounds, such as 7-ketocholesterol, can eventually be generated by oxidation of accumulated free and esterified cholesterol. Indeed, it has been shown in macaque monkeys that this oxysterol accumulates in the RPE/choroid and to a lower extent in the neural retina as a consequence of aging (80). Rodriguez et al. speculated that oxidized lipids could be carried from the neural retina back to the RPE via HDL-like particles (81). While cholesterol metabolism has been well documented in the RPE/choroid – Bruch’s membrane area during aging and AMD, it has been poorly studied so far specifically in the neural retina.

### 2.2 Evidence for neuron-glia cooperation in retinal cholesterol metabolism

As described in section 2.1, the retina exhibits a peculiar cholesterol metabolism. However, the relative contribution of each retinal cell type to the different aspects of this cholesterol metabolism is still not fully elucidated. Indeed, the retina is composed of a variety of cell types that are highly organized under a unique architecture. Information afforded by analyses performed *in vivo* or on whole tissue extracts does not document which cell types are implicated in cholesterol metabolism. In the present review, focus is put on Müller cells even though other macroglial cells and microglial cells also likely contribute to cholesterol metabolism in the retina (82). The data described below tend to support the concept of a neuron-glia cooperation for cholesterol metabolism in the retina as illustrated in **Figure 3**.

#### 2.2.1 Neurons depend on glial cholesterol

As mentioned in the Introduction, nervous tissues are made of neurons and glial cells interacting with each other. Neuron-glia cooperation at different levels is essential for CNS development and function. Pfrieger proposed the existence of a metabolic cooperation for cholesterol between these two cell types based on the observation that formation and activity of synapses of cultured neurons depend on a soluble factor secreted by macroglial cells, later identified as cholesterol in ApoE-containing LPs (83–85). He then made the hypothesis that, “neurons reduce or even abandon cholesterol synthesis to save energy and import cholesterol from astrocytes *via* lipoproteins” (86). This idea has since been confirmed by several study results. First, axon growth of neurons was promoted by glia-derived ApoE-containing LPs and glial cell-conditioned medium. This effect was inhibited by HMGCR inhibition in glial cells suggesting the importance of cholesterol in these LPs (87). However, the same research group later reported that synthetic LPs containing ApoE, phospholipids and sphingomyelin but no cholesterol also stimulated axonal extension unexpectedly suggesting that cholesterol might not be mandatory for the stimulatory effect of ApoE-containing LPs produced by glial cells. Second, the rate of cholesterol synthesis in primary cultured neurons from post-natal rats has been shown to be markedly lower than in glial cells, possibly because of a low capability of converting lanosterol (88). It should be noted that these data come from experiments using retinal ganglion cells as a neuron model suggesting that the hypothesis that neurons outsource cholesterol synthesis to glial cells applies to the retina. Third, in a conditional transgenic mouse model of cholesterol biosynthesis inactivation in specific cerebellar adult neurons, the authors reported normal development and no sign of neuronal degeneration showing that these neurons do not require cell-autonomous cholesterol synthesis for survival or function and suggesting that they meet their needs by uptake from their surroundings, likely glial cells (89). However, the dependency for exogenous cholesterol does not seem to affect developing neurons. Indeed, mutant mice with blocked cholesterol synthesis in neuronal precursor cells during embryonic development exhibited reduced brain size, apoptotic neurons and perinatal lethality showing that developing neurons need endogenous cholesterol production (90). Moreover, it has been reported *in vitro* that, at the developing stage, neurons, like astrocytes, actively synthesize cholesterol and rely on this endogenous biosynthesis for their survival and normal functioning. They appeared to accumulate most of their newly synthesized cholesterol while astrocytes secreted most of it. Developing neurons could also take up ApoE-complexed cholesterol from their local environment (91).

#### 2.2.2 Müller cells as cholesterol suppliers for the retina

It has been demonstrated that the retina is able to synthetize cholesterol *de novo* but the contribution of specific retinal cell types to this process remains unclear. Several lines of evidence suggest that Müller cells, the principal glial cells of the vertebrate retina, play the role of astrocytes in the brain, being in charge of cholesterol biosynthesis and its delivery to neurons.

While isolated primary cell cultures are highly simplified models that do not mirror the complex physiology of the retina, they are still informative regarding the features of a specific cell type. We performed a study on cholesterol metabolism in rat primary Müller cell cultures (92). Using RT-qPCR, we showed that Müller cells express various genes involved in cholesterol metabolism including synthesis (*Hmgcr*), uptake and export *via* LPs (*Ldlr*, *Srb1*, *ApoE* and *Abca1*), elimination *via* oxysterols (*Cyp27a1, Cyp46a1*). Several sterols could be measured using gas chromatography. While cholesterol was by far the most abundant sterol, significant amounts of precursors (zymosterol, desmosterol, lathosterol) were also detected indicating that cholesterol biosynthesis takes place in Müller cells. The robust gene expression of *ApoE* and its partner *Abca1* indicated that Müller cells could export the cholesterol they synthesize by releasing ApoE-containing LPs such as HDL-like LPs to deliver it to neurons. Moreover, the expression of *Ldlr* and *Srb1* genes suggested that Müller cells could be able to uptake and remodel exogenous LPs. The low expression of *Cyp27a1* and especially of *Cyp46a1* suggested that elimination of cholesterol *via* the oxysterol pathway is not a major function of Müller cells. Several other studies have been performed on primary Müller cell culture focusing on cholesterol metabolism. Characterization of the transcriptome of mouse primary Müller cells identified *ApoE* as a gene specific of Müller cells as it was highly enriched compared with RGCs or rod PRs (93). Amaratunga et al. used radiolabeled amino acids to track newly synthesized proteins in primary rabbit Müller cell culture. They could detect labeled ApoE in the culture medium indicating that these cells are able to synthesize and secrete this apolipoprotein (94). The same research group then reported that the ApoE produced by Müller cells is packaged into LPs (95). These LPs contained mainly esterified cholesterol and triglycerides, but also free cholesterol and diglycerides. On top of ApoE, they also contained ApoJ, thus differing from plasma LPs. Interestingly, these LPs exhibited similar features as the LPs found in the vitreous of rabbits following injection of radiolabeled amino acids into the vitreous. Since labeled ApoE was also detected within the optic nerve (94), these observations suggest that Müller cells do synthesize and secrete ApoE-containing LPs *in vivo* that can be internalized by RGCs and transported within the optic nerve. ABCA1 and ABCG1 have been shown to be expressed in mouse primary cultures of Müller cells (53) suggesting that the transporters participate into the lipidation of ApoE-containing LPs produced by these glial cells. Apolipoproteins and lipids could also be secreted independently and assembled in the extracellular medium or vitreous.

As mentioned above, the contribution of specific retinal cell types to cholesterol metabolism remains hard to determine *in vivo*. Immunohistochemistry could enable to partially get at that issue. However, localization to a specific retinal layer is not always sufficient since more than one cell type can reside in the same retinal layer. Besides, Müller cells are challenging since, contrary to most other cell types of the retina, which are localized to a specific retinal layer, they expand throughout the entire depth of the retina. If labeling does not show the specific radial fiber pattern evocative of this cell type, localization to Müller cells is difficult to assure. This was the case for experiments performed on human retina immunostained for proteins of the cholesterol metabolism, among which HMGCR, LDLR or ABCA1 (46). Immunoreactivity was detected in specific retinal layers. HMGCR and LDLR co-localized in the ganglion cell layer (GCL), the inner and outer nuclear layers, the inner and outer plexiform layers, the external limiting membrane, all of which are crossed by Müller cells, as well as in the PR inner segments and the RPE. However, astrocytes are also present in the GCL. It was shown on flatmounted mouse retinas that ABCA1 colocalized with signal for glial fibrillary acidic protein, a marker for astrocytes, and only weakly with vimentin, a marker for Müller cells suggesting that Müller cells are not the major ABCA1 expresser among retinal glial cells (65). In the rat, Fliesler reported expression of HMGCR in Müller cells, as well as in the RPE and the rod outer segments (41). In the monkey retina, LDLR, SRB-I and SRB-II, LCAT and CETP were also detected in GCL, which includes Müller cell processes (43) suggesting the Müller cells may uptake intraretinal LPs and participate in their remodeling. This was supported by a histological study in the rat showing LRP1 expression in Müller cells (58). Recently, expression of ApoJ in Müller cell somas and end feet was clearly shown on mouse retinal sections using glutamine synthetase colocalization to identify Müller cells and *Apoj* ^-/-^ mice as a negative control (55).

As described in section 2.1.1, beside endogenous *de novo* biosynthesis, retinal cholesterol also originates from circulating LPs. Cholesterol uptake takes place at the outer BRB where RPE cells are the major players in this process. However, some authors have suggested that Müller cells could also participate in lipid uptake from the circulation through a transcytosis of LDL particles from retinal capillaries in the neural retina (81). Indeed, Müller cells surround the tightly junctioned retinal capillaries and participate, together with the endothelial cells, in forming the inner BRB. This still speculative pathway might contribute to the supply of sterols to the retina.

#### 2.2.3 Neurons can uptake and eliminate cholesterol

Brain neurons express different members of the LP receptor family, LRP1 and LDLR (52), which ensure ApoE-containing LP endocytosis and supply of lipids. In the monkey retina, LDLR was detected in the GCL and the outer plexiform layer where horizontal cells and PRs form their synapses. SRBI and SRBII were also localized in the GCL and PR inner segment layer, suggesting that neurons uptake lipids from LPs in the intraretinal environment. The detection of CETP and LCAT in these areas indicates that neurons could also participate in the remodeling of LPs (43, 45). Interestingly, authors reported that retinal expression of ApoE coincide with Müller cell differentiation and neuronal membrane development. They observed a switch from ApoA-I/SRBI to ApoE/LRP1 expression pattern in parallel to retinal development and an increase in content and complexity of retinal lipids. LRP1, but not LDLR, was detected using immunofluorescence on the contours of many neurons and PRs in the developing retina while ApoE transcript was concentrated in Müller glial cells as shown by *in situ* hybridization (56) supporting the idea of neurons uptaking lipids from glial origin.

As mentioned in section 2.1.3, conversion to oxysterols by CYP enzymes is a major pathway of cholesterol efflux from the retina. The expression of the different CYP enzymes in different retinal layers has been studied in rodents, monkeys, and humans (26). CYP46A1 appears to be specific to RGCs but is also weakly expressed in RPE, while CYP11A1 and CYP27A1 appear to be more ubiquitous, including expression in the GCL and a strong expression in the inner segments of PRs (46, 96). On mouse retinal sections, CYP46A1 was shown to be expressed specifically in RGCs (and in some cells at the edge of the inner nuclear layer) (97). RGCs were also shown to produce 24S-OHC *in vitro* (88). Even though CYP46A1 expression seems to be quite specific to RGCs, it appears that elimination of cholesterol is not restricted to neurons in the retina, contrary to the brain. In the latter, the CYP46A1 enzyme, the major cholesterol hydroxylase, was shown to be almost exclusively expressed in neurons in multiple brain subregions, using *in situ* hybridization and immunohistochemistry (97, 98). In the retina, other cholesterol hydroxylases expressed in other cell types, especially Müller cells, are also implicated. It has been shown using immunohistochemistry in monkey retina that CYP27A1 is expressed in Müller cells (96). Transcripts were also measured in primary culture of rat Müller cells, as well as CYP46A1’s to a lower extent (92).

Brain neurons are known to express various membrane transporters, namely ABCA1 and ABCG1, as well as ABCG4 (51). They participate in the removal of excess cholesterol from neurons *via* LPs present in their environment that could then reach the circulation (99, 100). ABCA1 and ABCG1 have been shown to be expressed in primary cultures of RGCs but to a lesser extent than in Müller cells (53). In the human retina, ABCA1 was detected in the GCL (46, 101). ABCA1 and the bi-directional SRB receptors, as well as ApoA1 were detected in the GCL and inner segments of the PRs in monkey retinas (45). ABCA1 was also detected in the inner segments of the PRs in the mouse retina (44). Robust ApoJ expression was shown in PRs on mouse retinas (55). Those data suggest that retinal neurons can also efflux excess cholesterol via intraretinal LPs. The importance of this pathway in retinal cholesterol homeostasis and integrity has been demonstrated in a mouse model of rod-specific *Abca1/g1* KO (68). These exhibited age-related accumulation of cholesterol and oxysterols in the outer retina, PR dysfunction and degeneration of rod outer segments leading to blindness (see **Table 1**).

#### 2.2.4 Oxysterols might be regulators of neuron-glia cooperation

In the brain, neurons and astrocytes cooperate for cholesterol metabolism, which is segregated and specialized between the two cell types: glial cells mostly synthesize and release cholesterol while neurons mostly utilize and eliminate it. Evidence presented above suggests that this cooperation concept might also exist in the retina. This requires that neurons and glial cells interact to coordinate and maintain cholesterol homeostasis. These interactions must rely on signaling molecules and regulatory mechanisms.

Cholesterol homeostasis is maintained *via* a sterol-sensing system regulating the expression and/or activity of enzymes and proteins of the cholesterol biosynthesis pathway, uptake, trafficking and efflux. Two major families of transcription factors are involved: Liver X Receptors (LXRs) and Sterol Regulatory Element-Binding Proteins (SREBPs), which regulate the transcription of genes coding for enzymes of cholesterol metabolism. LXRs are associated with hypocholesterogenic mechanisms, activating *Abca1*, *ApoE*, while SREBPs are associated with hypercholesterogenic mechanisms, activating *Hmgcr* or *Ldlr*. Cholesterol itself performs the central role in maintaining its own homeostasis since it can directly regulate the expression of enzymes of its own metabolism through inactivation of SREBP-2. Some cholesterol precursors, namely desmosterol and zymosterol, have been identified as LXR ligands, contrary to cholesterol itself (102). As mentioned in the Introduction (section 1.1), oxysterols, such as 24S-OHC and 27-OHC, can also bind and activate LXRs. Oxysterols can inhibit SREBPs activation by binding to INSIG (insulin-induced gene protein), a regulatory protein for this transcription factor. They also bind Oxysterol Binding Proteins (OSBPs), which are involved in lipid metabolism (8). Oxysterols thus are not only elimination products of cholesterol but also signaling molecules. It was therefore proposed that the neuron-specific oxysterol 24S-OHC is responsible for an interplay between neurons and astrocytes in the brain: it is released by neurons to inform astrocytes of their cholesterol status and glial cells consecutively adjust their cholesterol metabolism to meet neuron needs (86, 100). This idea relies on the fact that 24S-OHC production by CYP46A1 is mainly determined by the availability of its substrate, cholesterol, since the enzyme does not seem to undergo major regulation, and that 24S-OHC can freely diffuse from neurons up to surrounding astrocytes. 24S-OHC (1-10 µM, 24-72h) has been shown to stimulate ABCA1 and ApoE expression in a dose and time-dependent manner in brain astrocytes suggesting it promotes cholesterol efflux in these cells (103). Moreover, activation of LXR, whose oxysterols are ligands, was shown to result in increased cholesterol release from glial cells (104). Moreover it was shown that 24S-OHC (10µM, 24h) down-regulates the expression of enzymes of the cholesterol biosynthesis pathway including HMGCR, while up-regulating the expression of ApoE in rat cortical neurons suggesting that it also regulates cholesterol metabolism in neuronal cell types (105).

**Figure 4:**
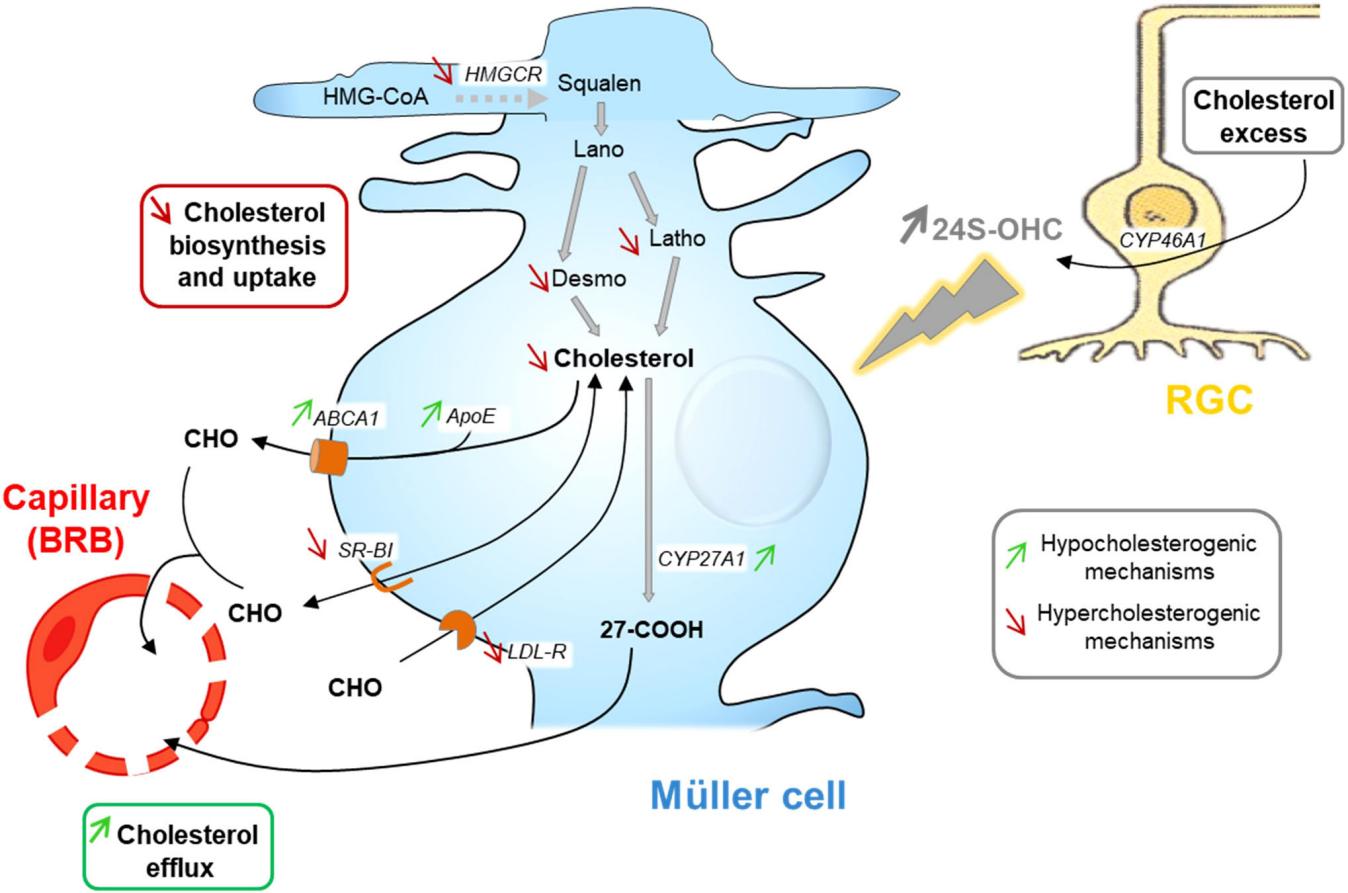
24S-OHC as a signaling molecule in neuron-glia interaction in the retina: schematic representation of its regulatory effect on Müller cell cholesterol metabolism and its hypothetical role in maintaining retinal cholesterol homeostasis. 24S–OHC (24(S)-hydroxycholesterol) exposure leads to strongly decreased cholesterol content in Müller cell cultures. This occurs through decreased cholesterol synthesis, via *HMGCR* (HMG-CoA reductase) gene downregulation and reduction of cholesterol precursor amounts, increased cholesterol efflux, suggested by *Abca1* (ATP-binding cassette transporter A1), *ApoE* and *Cyp27a1* (cytochrome P450 family 27 subfamily A member 1) gene upregulation, and decreased lipoprotein uptake via down-regulation of *Ldlr (*low density lipoprotein receptor) and *SrbI* (scavenger receptor class BI) gene expression. Considering those regulations, we hypothesize that retinal ganglion cells release 24S-OHC when their cholesterol levels are high. Müller cells then respond to these increased 24S-OHC levels by diminishing their cholesterol biosynthesis and increasing efflux through the systemic circulation in order to adjust retinal cholesterol levels to neuron needs and thus maintain retinal cholesterol homeostasis. ABCA1, ATP-binding cassette transporter A1; Apo, apolipoprotein; BRB, blood-retina barrier; CHO, cholesterol; CYP27A1, cytochrome P450 family 27 subfamily A member 1; CYP46A1, cytochrome P450 family 46 subfamily A member 1; Desmo, desmosterol; HMG-CoA, 3-hydroxy-3-methylglutaryl-CoA; HMGCR, HMG-CoA reductase; Lano, lanosterol; LDLR, low density lipoprotein receptor; Latho, lathosterol; RGC, retinal ganglion cell; SRB, scavenger receptor class BI; 24S-OHC, 24S-hydroxycholesterol; 27-COOH, 3β-hydroxy-5-cholestenoic acid. Figure adapted from (92); Copyright Elsevier 2023.

Regarding the retina, we showed, using primary rat Müller cell cultures, that these retinal glial cells adjust their cholesterol metabolism in response to treatment with 24S-OHC (0.5-1.5 µM, 48h) (92). 24S-OHC exposure induced a range of pathways resulting in cholesterol depletion. These included a decrease in cholesterol biosynthesis as illustrated by decreased levels of cholesterol precursors, desmosterol and lathosterol, and a lower expression of *Hmgcr* gene. These also included decreased *Ldlr* and *Srb1* gene expression suggesting a diminished LP uptake. The gene expression of *Cyp27a1*, in charge of cholesterol elimination via oxysterol production, was slightly increased. *Abca1* and *ApoE* expression were also increased suggesting an increased cholesterol efflux. As proposed by Pfrieger in the brain, this could correspond to the secretion of specific LPs by glial cells in response to neuron-derived 24S-OHC that cannot be taken up by neurons thereby reducing retinal cholesterol levels (86). Since 24S-OHC is a molecule produced by neurons, especially RGCs, depending on the availability of CYP46A1 substrate, cholesterol, our observations in primary Müller cell cultures thus suggest that, as in the brain, 24S-OHC could be a mediator of neuron glia communication underlying the cooperation between these two cell types for cholesterol homeostasis. Müller cells being responsive, at least in culture, to very low doses of 24S-OHC, this molecule may be implicated in a fine regulation mechanism. Experimental data detailed above and showing the regulatory effect of 24S-OHC on Müller cell cholesterol metabolism as well as its proposed role in modulation of cholesterol levels in the retina implicating neuron-glia interactions are illustrated in **Figure 4**.

The data detailed in this section indicate that the requisite components of a neuron-glia cooperation for cholesterol metabolism are present in the retina. Especially, the Müller cells, with their bipolar shape and long process directly contacting all nerve cells across the entire retina and with their active cholesterol metabolism, are ideally suited to provide cholesterol to meet neuron needs. It is tempting to consider that cholesterol homeostasis in the retina, as in the brain, relies on this cellular cooperation and that oxysterols play a signaling role between neurons and glial cells. Further investigations are however required to better define this concept.

## 3 Cholesterol, oxysterols and glaucoma

Studies are still underway to improve our knowledge of glaucoma pathophysiology. Several molecular mechanisms have been proposed to explain the development of the disease (see section 1.3). Although it remains an understudied pathway, data from both observational studies in humans and laboratory experiments that support a contribution of cholesterol metabolism in glaucoma pathogenesis, are presented in this section.

### 3.1 Clinical data

#### 3.1.1 Epidemiological links with cholesterolemia

Circulating cholesterol levels undergo changes during aging, one of the main risk factors for glaucoma. Especially, serum cholesterol and LDL-C have been shown to decline with aging, associated with both decreased dietary absorption and hepatic synthesis (78). Epidemiological studies have investigated potential associations between blood total cholesterol, LDL-C or HDL-C levels and different types of glaucoma. Although they provide valuable information, they do not permit, especially cross-sectional studies, to infer causal relationship between the investigated parameters and disease. The design and main results of selected studies are summarized in **Table 2** for POAG and **Table 3** for other types of glaucoma.

**Table 2:** Cholesterolemia and POAG – Epidemiological links from observational studies.

Author (Year)	Study Design	Study Population (Age mean± SD)	Findings/Results
Elisaf et al. (2001) (106)	Case-control	72 controls (63 ± 8) 49 cases (65 ± 9) Greece	No significant difference between patients and controls in serum CHO (233.7 ± 45.9 *vs* 237.5 ± 49.0 mg/dl, NS), HDL-C (50.6 ± 14.9 vs 51.8 ± 14.4 mg/dl, NS), or LDL-C (160 ± 36 *vs* 156 ± 42mg/dl, NS)
Ishikawa et al. (2011) (107)	Cross-sectional	710 controls (53 ± 10) 29 cases (59 ± 11) Japan	No significant difference between patients and controls in CHO (211.0 ± 31.2 *vs* 211.1 ± 33.2mg/dl, *p*=0.90)
Wang et al. (2012) (108)	Cross-sectional	2,951 controls 71 PAOG (median=60) China	No significant association between POAG and high serum CHO (OR=0.95, 95%CI=0.74-1.22; *p*=0.70), low HDL-C (OR=0.84, 95%CI=0.40-1.73; *p*=0.63), or high LDL-C (OR=0.98, 95%CI=0.71-1.23; *p*=0.91)
Modrzejewska et al. (2015) (109)	Case-control	54 controls (68 ± 5 56 cases (68 ± 0) Poland	Higher plasma CHO in patients (200.2 ± 22.09mg/dL) *vs* controls (165.2 ± 44.9mg/dL) (*p*=0.000) Higher plasma LDL-C in patients (133.1 ± 30.14mg/dL) *vs* controls (83.2 ± 26.6mg/dL) (*p*=0.000) Lower plasma HDL-C in patients (38.3 ± 4.07mg/dL) *vs* controls (44.1 ± 14.3mg/dL) (*p*=0.004)
Kim et al. (2016) (110)	Cross-sectional	4,062 controls (40 ± 0) 124 cases (50 ± 2) South Korea	Lower serum HDL-C in patients (47.2 ± 1.1mg/dL) *vs* controls (50.8 ± 0.2mg/dL) (*p*=0.001)
Huang et al. (2022) (111)	Meta-analysis (2 cross-sectional and 3 case-control studies)	23,296 controls 1,315 cases China, South Korea	No significant association between POAG and serum CHO (OR=1.00, 95%CI=0.99-1.01) Negative association between POAG and HDL-C (OR=0.96, 95%CI=0.94-0.99)
Joshi & Adatiya et al. (2023) (112)	case-control	50 controls (60 ± 9) 50 cases (63 ± 10) India	Higher serum CHO in patients (205.2 ± 36.9mg/dL) *vs* controls (177.7 ± 22.6mg/dL) (*p<*0.001) Higher serum LDL-C in patients (139.50 ± 31.03mg/dL) *vs* controls (114.9 ± 17.7mg/dL) (*p<*0.001) No significant difference between patients and controls in HDL-C (35.8 ± 9.6 *vs* 37.2 ± 6.2 mg/dl, *p*=0.380)
Yang et al. (2023) (66)	case-control	100 controls 100 cases China	Lower serum HDL-C in patients (1.26mmol/L ± 31.03mg/dL) *vs* controls (1.46mmol/L ± 17.7mg/dL) (*p<*0.0001)

Results are given as mean ± SD (except for (110) mean ± SE) or OR, 95%CI. See references for details regarding specific statistical methods. CHO, cholesterol; CI, confidence interval; LDL-C, low density lipoprotein cholesterol; HDL-C, high density lipoprotein cholesterol; OR, odds ratio; POAG, primary open angle glaucoma; NS, non-significant; NTG, normal tension glaucoma. In (107) and (110), age was significantly different between controls and cases.

**Table 3:** Cholesterolemia and other glaucoma types – Epidemiological links from observational studies.

Author (Year)	Study Design	Glaucoma type	Study Population (Age mean ± SD/SE)	Findings/Results
Su et al. (2006) (113)	Case-control	NTG	40 controls (49 ± 12) 40 cases (50 ± 12) Taiwan	No significant difference between patients and controls in serum CHO (183 ± 32 *vs* 180 ± 31mg/dL, *p*=0.721), HDL-C (53 ± 14 *vs* 52 ± 11mg/dL, *p*=0.766), or LDL-C (107 ± 28 *vs* 114 ± 29mg/dL, *p*=0.323)
Yuki et al. (2010) (114)	Case-control	NTG	40 controls (62 ± 15) 43 cases (59± 10) Japan	No significant difference between patients and controls in serum CHO (224.5 ± 46.7 *vs* 216.0 ± 39.6 mg/dl, *p*=0.38)
Yüksel et al. (2010) (115)	Case-control	PEXG	25 controls (65 ± 5) 26 cases (66 ± 9) Turkey	No significant difference between patients and controls in serum CHO (183.7 ± 21.0 *vs* 183.8 ± 15.7mg/dL, *p*=0.097), HDL-C (49.8 ± 10.6 *vs* 50.4 ± 11.4mg/dL, *p*=0.956), or LDL-C (109.4 ± 22.7 *vs* 111.6 ± 25.1mg/dL, *p*=0.461)
Kim et al. (2014) (116)	Cross-sectional	NTG	17,940 controls (53 ± 8) 300 cases (54 ± 8) Korea	No significant difference between patients and controls in serum HDL-C (50.0 ± 11.3 *vs* 50.2 ± 11.9mg/dL, *p*=0.708 for males; 59.5 ± 14.8 *vs* 59.6 ± 14.0mg/dL, *p*=0.823 for females)
Kim et al. (2014) (117)	Cross-sectional	NTG	4015 controls (31 ± 6) 80 cases (32 ± 5) Korea	No significant association between NTG in young people and plasma CHO (OR=1.0, 95%CI=0.99-1.00, *p*=0.829) Significant association between NTG in young people and low plasma HDL-C (OR=0.96, 95%CI=0.94-0.99, *p*=0.004)
Türkyilmaz et al. (2014) (118)	Case-control	PEXG	25 controls (64 ± 7) 25 cases (65 ± 7) Turkey	No significant difference between patients and controls in serum CHO (191.2 ± 22.1 *vs* 181.8 ± 26.8mg/dL, *p*=0.187), HDL-C (40.6 ± 12.1 *vs* 46.3 ± 15.2mg/dL, *p*=0.151), or LDL-C (129.6 ± 26.1 *vs* 115.8 ± 34.7mg/dL, *p*=0.118)
Bossuyt et al. (2015) (119)	Case-control	NTG	33 controls (67 ± 8) 30 cases (65 ± 8) Netherlands	No significant difference between patients and controls in serum CHO (201 ± 34 *vs* 215 ± 30mg/dL, *p*=0.08), HDL-C (69 ± 16 *vs* 75 ± 21mg/dL, *p*=0.18), or LDL-C (111 ± 28 *vs* 120 ± 32mg/dL, *p*=0.25)
Ko et al. (2016) (120)	Cross-sectional	Glaucoma (CDR≥0.6 at least one eye)	5,574 controls (56 ± 0) 172 cases (68 ± 1) United States	No significant association between glaucoma and high CHO (OR=0.69, 95%CI=0.42-1.11), low HDL-C (OR=1.04, 95%CI=0.61-1.77), or high LDL-C (OR=1.29, 95%CI=0.56-2.98)
Yilmaz et al. (2016) (121)	Case-control	NTG/PEXG	40 controls (55 ± 13) 32 NTG (60 ± 19) - 31 PEXG (63 ± 11) Turkey	Higher serum CHO in NTG (221.5 ± 56) *vs* PEXG (209.4 ± 47mg/dL) and controls (191 ± 39mg/dL) (*p*=0.01) No significant difference between patients and controls in HDL-C or LDL-C
Shao et al. (2021) (122)	Case-control	PACG	242 controls (58.9 ± 8.88) 320 cases (60.5 ± 11.8) China	Higher serum levels in patients *vs* controls in CHO (4.71 ± 1.01 *vs* 4.10 ± 0.77mmol/L, *p*<0.001), LDL-C (2.67 ± 0.79 *vs* 2.51 ± 0.58mmol/L, *p*=0.005), and HDL-C (1.21 ± 0.34 *vs* 0.96 ± 0.21mmol/L, *p*<0.001)
Posch‐Pertl et al. (2022) (123)	Meta-analysis (26 studies)	PEXG, POAG, NTG	350,441 controls – 7,196 cases Netherlands, Serbia, South Korea, Turkey, USA, Poland, Bosnia and Herzegovina, Iran, Taiwan, Japan, UK	Higher CHO in patients *vs* controls (MD=7.9mg/dL, 95%CI=3.3-12.5; *p* = 0.001) Lower HDL-C in patients *vs* controls (MD=-2.0mg/dL, 95%CI=-3.1–0.9; *p* = 0.001) No significant difference between patients and controls in LDL-C (MD=6.1mg/dL, 95%CI=-4.3-16.4; *p* = 0.251)

While NTG is a subtype of POAG, it exhibits differences raising questions about specific pathological mechanisms. Studies focusing on NTG have therefore been separated from POAG (**Table 2**) and included in this table. Results are given as mean ± SD (except for (120) mean ± SE) or OR, 95%CI. See references for details regarding specific statistical methods. CHO, cholesterol; CI, confidence interval; LDL-C, low density lipoprotein cholesterol; HDL-C, high density lipoprotein cholesterol; NTG, normal tension glaucoma; OD, odds ratio; PACG, primary angle-closure glaucoma; POAG, primary open angle glaucoma; PEXG, pseudoexfoliation glaucoma; PACG, primary angle-closure glaucoma; CHO, cholesterol; HDL-C, High density lipoprotein; LDL-C, Low density Lipoprotein; MD, mean difference.

Overall, results are quite conflicting. Some studies reported higher circulating cholesterol levels in POAG patients compared with control subjects (109, 112) while other studies failed to measure any difference (106–108, 111). Regarding normal-tension glaucoma (NTG), a subtype of POAG, most studies reported no difference in cholesterol levels between patients and controls (113, 114, 117, 119) while one study reported higher cholesterol levels in patients (121). For pseudoexfoliation glaucoma (PEXG), studies consistently found no difference in cholesterol levels between patients and controls (115, 118). One study reported higher cholesterol levels in primary angle-closure glaucoma (PACG) patients compared with controls (122). Interestingly, no study observed lower circulating cholesterol in glaucoma patients. Regarding HDL-C, some studies reported lower levels in POAG (66, 109–111) or young NTG patients (117) compared with controls while others reported no difference (106, 108, 112, 113, 115, 116, 118–121) in different glaucoma types. Apart from a study on PACG (122), no study reported higher HDL-C in glaucoma patients. Conflicting results on the possible link between HDL-C and glaucoma or IOP have been summarized in a literature review of epidemiological studies focusing on HDL-C and age-related ocular diseases (124). Regarding LDL-C, disparities in the results of observational studies also exist. Some reported higher levels in POAG (109, 112) and PACG (122) patients compared with controls while others reported no difference (106, 108, 113, 115, 118–121) in different glaucoma types. No study reported lower LDL-C in glaucoma patients. A recent and comprehensive review included 26 studies in a random-effects meta-analysis (123). Results showed that circulating cholesterol levels were higher in glaucoma patients (all types) compared with controls (Mean difference 7.9 mg/dl, 95% CI 3.3 to 12.5, *p* = 0.001). LDL-C tended to be higher but the difference did not reach statistical significance, and HDL-C levels were lower (Mean difference -2.0 mg/dl, 95% CI: -3.1 to -0.9, *p* = 0.001). While these observations support a role for cholesterolemia in glaucoma, interstudy heterogeneity was substantial and causality cannot be presumed, as mentioned by the authors.

Studies focused on the use of statins (HMGCR inhibitors), which are widely used medications for treating hypercholesterolemia and preventing stroke and coronary artery disease, but its association with glaucoma remains controversial. Several studies reported that statin use, especially long-term, was associated with a lower risk of POAG (125–128). On the other hand, a recent study found no association between short-term statin use and onset of POAG in dyslipidemic Japanese subjects (129) while a positive association between statin use, especially high dosage, and POAG was found in a Taiwanese population (130). A meta-analysis that included seven cohort studies, three case-control studies, and one cross-sectional study for a total of 583,615 participants demonstrated a statistically significant association between a short-term statin use (less than 2 years) and a reduced incidence of glaucoma, independently of IOP, while longer use showed no association (131).

#### 3.1.2 Epidemiological links with gene variants

Several genetic association studies have been conducted to investigate a potential link between variants of genes implicated in cholesterol metabolism and glaucoma. Those studies could indicate that systemic levels of cholesterol can affect the disease. However, most of these genes are expressed locally in the retina and the possibility that mechanisms underlying the effects of these genetic variations might stand in local retinal cholesterol metabolism cannot therefore be excluded. On the other hand, such studies do not permit to ascertain an implication of cholesterol metabolism in the considered disease since the players investigated are frequently known to have functions other than regulation of cholesterol metabolism. Selected studies are summarized in **Table 4**.

**Table 4:** Variants in genes of cholesterol metabolism and glaucoma – Epidemiological links from genetic association studies.

Genes	rs	Authors	Glaucoma type	Study population	Findings (*p*; OR (95% CI))
***Abca1***	2164560	Chen et al. (2014) (101)	POAG	2,906 controls – 5,974 cases China, Singapore (meta-analysis)	Negative association (*p=*5.66x10^-10^; 0.78 (0.73-0.85))
	2422493	Luo et al. (2015) (132)	PACG	1,311 controls – 1,122 cases China	No significant association
		Wang et al. (2019) (133)	PACG	720 controls – 500 cases China	No significant association
	2472459	Chen et al. (2014) (101)	POAG	2,906 controls – 5,974 cases China, Singapore (meta-analysis)	Negative association (*p=*1.47x10^-11^; 0.77 (0.72-0.83))
		Luo et al. (2015) (132)	PACG	1,311 controls – 1,122 cases China	No significant association
		Wang et al. (2019) (133)	PACG	720 controls – 500 PACG China	No significant association
	2472493	Gharahkhani et al. (2014) (134)	POAG	11,488 controls – 4,703 cases Australia, USA (meta-analysis)	Positive association (*p*=2.1x10^-19;^ 1.31)
		Hysi et al. (2014) (135)	POAG	95,560 controls – 4,284 cases Multiethnic (meta-analysis)	Positive association (*p*=4.15x10^-9^; 1.24 (1.16-1.34))
		Luo et al. (2015) (132)	PACG	1,311 controls – 1,122 cases China	No significant association
		Bonnemaijer et al, (2018) (136)	POAG	1,826 controls – 1,113 cases Africa, USA (meta-analysis)	No significant association
		Choquet et al. (2018) (137)	POAG	58,426 controls – 4,986 cases Multiethnic	Positive association (*p=*1.2x10^-13^; 1.17 (1.12-1.22)
		MacGregor et al. (2018) (138)	POAG	119,318 controls –7,947 cases Multiethnic (meta-analysis)	Negative association (*p*=4.30x10^-30^; 0.84 (0.80-0.87))
		Shiga et al. (2018) (139)	POAG/HTG/NTG	17,570 controls – 3,398 POAG 18,815 controls – 808 HTG – 1,026 NTG Japan	Positive association POAG: *p*=1.45x10^-9;^ 0.84 (0.80-0.89), HTG: *p*=4.95x10^-4;^ 0.82 (0.75-0.90), NTG: *p*=2.11x10^-3;^ 0.85 (0.79-0.93)
		Wang et al. (2019) (133)	PACG	720 controls – 500 cases China	No significant association
		Kondkar et al. (2022) (140)	PACG/PEXG	246 controls – 102 PACG – 94 PXG Saudi Arabia	No significant association
		Araki et al. (2022) (141)	POAG	504 controls – 505 cases Brazil	No significant association
	2472494	Gharahkhani et al. (2021) (142)	POAG	349,321 controls – 34,179 cases Multiethnic (meta-analysis)	Positive association (*p*=9.38x10^-83^; 1.19)
	2472496	Luo et al. (2015) (132)	PACG	1,311 controls – 1,122 cases China	No significant association
		Wang et al. (2019) (133)	PACG	720 controls – 500 PACG China	No significant association
	2472519	Chen et al. (2014) (101)	POAG	2,906 controls – 5,974 cases China, Singapore (meta-analysis)	Negative association (*p=*2.68x10^-11^; 0.78 (0.72-0.84))
		Luo et al. (2015) (132)	PACG	1,311 controls – 1,122 cases China	No significant association
		Wang et al. (2019) (133)	PACG	720 controls – 500 PACG China	No significant association
	2487032	Chen et al. (2014) (101)	POAG	2,906 controls – 5,974 cases China, Singapore (meta-analysis)	Negative association (*p=*2.79x10^-19^; 0.73 (0.68-0.78))
		Luo et al. (2015) (132)	PACG	1,311 controls – 1,122 cases China	No significant association
		Wang et al. (2019) (133)	PACG	720 controls – 500 PACG China	No significant association
	2487042	Luo et al. (2015) (132)	PACG	1,311 controls – 1,122 cases China	No significant association
		Wang et al. (2019) (133)	POAG	720 controls – 500 PACG China	No significant association
***ApoE***	ℇ4	Wang et al. (2013) (143)	POAG	1,756 controls – 1,916 cases Multiethnic (meta-analysis)	No significant association
		Song et al. (2013) (144)	POAG	1,793 controls – 1,928 cases Multiethnic (meta-analysis)	No significant association
		Liao et al. (2014) (145)	POAG	1,756 controls – 1,971 cases Multiethnic (meta-analysis)	Positive association for Asians (*p=*0.04; 3.55 (1.06-11.87)) No significant association for Caucasians
		Wang et al. (2014) (146)	All types	1,549 controls – 1,704 cases Multiethnic (meta-analysis)	Positive association for Asians (*p=*0.002; 5.22 (1.85-14.68)) No significant association for Caucasians
		Margeta et al. (2020) (147)	POAG	2,606 controls – 2,606 cases USA	Negative association (*p=*0.0022; 0.83 (0.74-0.94))
	449647	Chen et al. (2019) (148))	POAG	1,958 controls – 1,937 cases Multiethnic (meta-analysis)	Positive association (*p=*0.001; 1.33 (1.13-1.57))
***ApoJ***	2279590	Krumbiegel et al. (2009) (149)	PEXG	532 controls – 592 cases Germany, Italy	Positive association for Germans (*p=*0.0260; 1.26 (1.03-1.55)) No significant association for Italians
		Padhy et al. (2014) (150)	PEXG	89 controls – 55 cases India	Positive association (*p=0,031*; 1.73 (1.07-2.79))
		Fan et al. (2015) (151)	PEXG	446 controls – 177 cases US, Israël	No significant association
	3087554	Krumbiegel et al. (2009) (149)	PEXG	532 controls – 592 cases Germany, Italy	No significant association
		Padhy et al. (2014) (150)	PEXG	89 controls – 55 cases India	No significant association
		Fan et al. (2015) (151)	PEXG	446 controls – 177 cases US, Israël	No significant association
***Cyp46a1***	754203	Fourgeux et al. (2009) (152)	POAG	118 controls – 150 cases France	Positive association (*p*<0.05; 1.26 (1.006-1.574))
		Mossbock et al. (2011) (153)	POAG	251 controls – 330 cases Austria	No significant association
		Chen et al. (2012) (154)	POAG	577 controls – 462 cases China	No significant association
		Chandra et al. (2016) (155)	POAG	112 controls – 122 cases North India	Negative association (*p*<0.047; 0.6 (0.3-0.9))

Results are given as p-value; OR (95% CI). See references for details regarding specific statistical methods. CI, confidence interval; OR, odds ratio; POAG, primary open angle glaucoma; PACG, primary angle-closure glaucoma; PEXG, pseudoexfoliation glaucoma; NTG, normal-tension glaucoma; HTG, high-tension glaucoma. For ApoE, studies included in the 3 meta-analyses were not individually listed in the Table.

*Abca1* has been the most studied gene of cholesterol metabolism in the context of glaucoma. ABCA1 is a transporter required for the biosynthesis of HDL particles and a crucial regulator of cholesterol efflux from peripheric tissues to the liver *via* the systemic circulation. As mentioned earlier, it is active in the CNS and retina. Three meta-analyses including large numbers of subjects have positively associated POAG with rs2472493, located upstream of *Abca1*, in populations from various ethnicities and origins (134, 135, 137). By contrast, another large multi-ethnic meta-analysis reported a negative association (138). Studies in Brazilian, Afroamerican, Chinese, and Arab populations failed to detect any association between this *Abca1* polymorphism and different types of glaucoma (132, 133, 136, 140, 141). Interestingly, it was recently shown that this rs2472493 genetic variant attenuates *Abca1* expression in a model of cultured cells using a luciferase reporter gene under the control of a human *Abca1* promoter harboring the POAG risk allele [G] (66). This suggests that this genetic variant mediates transcriptional regulation of the *Abca1* gene, and that the diminished expression of *Abca1* might be a risk factor for POAG. Authors confirmed this idea in POAG patients from a small Chinese cohort who had lower *Abca1* mRNA levels compared with healthy subjects. Moreover, HDL-C levels were significantly lower in the POAG patients, which is consistent with ABCA1 being a player in HDL biogenesis. Also, POAG patients homozygous for the risk allele had lower *Abca1* mRNA levels and lower HDL-C levels compared with patients carrying the non-risk allele. A very large multiethnic meta-analysis recently showed a positive association between POAG and rs2472494 (142). The investigation of other SNPs of *Abca1* (rs2164560, rs2422493, rs2472459, rs2472496, rs2472519, rs2487032, rs2487042), mainly in Chinese populations, revealed no or negative association (101, 132, 133). In one of the studies, while no significant association was found between the disease and single polymorphisms, associations were shown for specific haplotypes combining several of these rs (132). However, these data regarding *Abca1* polymorphism do not prove that cholesterol metabolism is implicated in the pathogenesis of glaucoma since ABCA1 has been shown to play a role in IOP regulation by modulating Caveolin1/eNOSynthase/NO signaling pathway (156).

The study of the genetic association between *ApoE* and POAG has provided conflicting results as well. Two meta-analyses compiling data from mostly small size studies found no association between the *Apoϵ4* variant and POAG (143, 144). Two other meta-analyses (including almost the same studies) concluded that this variant is a risk factor for glaucoma specifically in Asians (145, 146). On the contrary, Margeta et al. reported a lower risk of POAG in Apoϵ4 carriers in a cohort of more than five thousand American subjects. The association was even more significant in NTG suggesting a role for ApoE in regulating RGC degeneration rather than IOP (147). Again, this is not a direct proof of the contribution of cholesterol metabolism to the pathological mechanisms of glaucoma since ApoE protein is known to fulfill functions in the CNS in addition to cholesterol transport. Indeed, a potential mechanism linking ApoE4 and glaucoma *via* Galectin-3 signaling was proposed by the authors. They reported that mouse models with *Apoe*-deficient or *Apoϵ4* expressing retinal microglia were protected against RGC degeneration and suggested that Apoe controls the microglial transition from a homeostatic to a cytotoxic neurodegenerative molecular phenotype implicating the immunoregulatory lectin, Galectin-3 (157). Besides, in the context of AD, it has been shown that ApoE is able to bind the amyloid-β (Aβ) peptide and form a complex that can interact with ApoE receptors resulting in Aβ peptide clearance. This could also be relevant for glaucoma since it is thought that Aβ peptide could play a role in the pathogenesis of this disease (158, 159).The Apoϵ4 isoform is much less efficient than the most common isoform Apoϵ3 to perform this function. This likely explains why the *Apoϵ4* allele, coding for the ApoE4 isoform, is the most important genetic risk factor known for AD (160). Interestingly, the *Apoϵ4* allele seems to be associated with a lower risk of AMD, another retinal neurodegenerative disease (161). Another apolipoprotein, ApoJ, has also been investigated in small cohorts of various origins for its association with PEXG. A positive (149, 150) or no association (151) was found for rs2279590 and no association with the disease was found for rs3087554 (149–151).

We reported for the first time an association between a *Cyp46a1* gene variant (rs754203) and increased risk of glaucoma in a French population (152). However, the consequences of this genetic variation on CYP46A1 enzyme protein levels and activity is unknown since plasma 24S-OHC levels were not different between the different genotypes or between glaucoma patients and controls. This genetic association was not confirmed in a larger cohort with similar ethnicity (153) and in a Chinese cohort (154), while a negative association has been reported more recently in an Indian cohort (155). A meta-analysis including the 3 first studies found no association (148). Interestingly, the same gene variant of *Cyp46a1* has been linked to an increased risk of AD, which shares some features with glaucoma (162–166).

The disparities in the results of the epidemiological association studies presented above may be attributed to different factors. POAG is a complex multifactorial disease with several genetic and environmental factors interplaying, and each single nucleotide variant of the different genes considered, as well as cholesterolemia, likely explain only a minor part of the disease. Studies are performed in various populations and ethnicities, which greatly influence physiology and the susceptibility of a population to a disease or a risk allele. Even though confounding variables are commonly adjusted for in statistical models, their selection can induce disparities in study results. Especially, the age difference between studies can be significant while age is a major risk factor for glaucoma. Aged people are usually under various medications, which can represent major bias. Finally, the small sample size of some studies represents a limitation for detecting fine associations.

### 3.2 Laboratory data

Few experimental data documenting the potential role of cholesterol metabolism in glaucoma are available.

The specific expression of CYP46A1 in RGCs, the cellular target of glaucoma, points toward a possible role of the enzyme and its product 24S-OHC in this disease. Ishikawa et al. studied an *ex vivo* model of glaucoma using rat eyecups maintained in a closed pressure-loading system. They reported that pressure elevation resulted in increased retinal CYP46A1 gene and protein expression within 24 hours. Enhanced expression was mainly observed in the RGC layer using immunofluorescence. It was associated with increased 24S-OHC levels and decreased cholesterol levels. Interestingly, CYP46A1 inhibition by voriconazole induced severe retinal damage, including in the RGC layer, which could be prevented by treatment with 24S-OHC. Moreover, exposure of the *ex vivo* rat eyecups to 24S-OHC was protective against axonal injury and apoptotic death of RGCs induced by elevated pressure. These findings suggest that the observed CYP46A1 overexpression induced by pressure elevation might be a restorative rather than a pathological mechanism (9). *In vivo*, we showed that laser-induced intraocular hypertension, the main risk factor for glaucoma, was associated with changes in oxysterol metabolism in the rat retina. Significant increases in the expression of CYP46A1 as well as in the levels of its product 24S-OHC were measured. The increase in CYP46A1 levels was early (3 days post-laser) and transient since it returned close to control levels 14 days post-laser (36). To further understand the link between CYP46A1 and retinal integrity, the effects of voriconazole were studied (167). Repeated intraperitoneal injections of voriconazole reduced retinal 24S-OHC levels and it was accompanied by an important reduction of the amplitude of ERG b-wave and oscillatory potentials indicating impaired function of the inner retina. The amplitude of pSTR (Scotopic Threshold Response) was also reduced suggesting RGC dysfunction. In this model, the retinal expression of CYP46A1 was slightly increased likely as a compensation of its decreased activity induced by voriconazole. Glial reactivity was also observed. It was proposed to be a response of glial cells to the decreased 24S-OHC levels in the context of a neuron-glia crosstalk. However, no significant modification of cholesterol levels could be measured and the observed effects of CYP46A1 inhibition by voriconazole on retinal function couldn’t therefore be attributed to a significant excess cholesterol consecutive to a decreased elimination. Since glaucoma presents common features with neurodegenerative diseases of the CNS, it is interesting to consider that apoptotic neuronal death was also observed in the hippocampus of mice with CYP46A1 inhibition induced by a ShRNA-coding AAV targeting neurons. It was associated with decreased 24S-OHC levels and increased cholesterol content in neurons (24). Recruitment of the amyloid precursor protein in lipid rafts and Aβ peptide production were also observed and attributed to neuronal cholesterol accumulation by the authors since cholesterol has been shown previously to facilitate these events. Decreased levels of CYP46A1 have been measured in different brain regions of patients with AD (168, 169) or HD (23), which likely results in a decreased cholesterol turnover. Besides, a positive effect of 24S-OHC on neuronal survival has been shown *in vitro* (10, 12). It is proposed that CYP46A1 upregulation *via* ROS accumulation during aging results in decreased cholesterol levels in neuron plasma membrane, via 24S-OHC efflux, favoring the recruitment of TrkB and the activation of the PI3K/Akt pro-survival pathway (12). El-Darzi et al. demonstrated decreased 24S-OHC levels in the retina of ApoJ KO mice that was associated with decreased retinal cholesterol levels and biosynthesis, and several features of glaucoma: increased IOP and cup-to-disk ratio, and impaired RGC functionality. Interestingly, treatment of ApoJ KO mice with efavirenz, an activator of CYP46A1, enabled restoration of retinal 24S-OHC and cholesterol levels as well as physiologic IOP and RGC functionality. The underlying mechanisms did not seem to implicate glial cells since the authors reported no activation of Müller cells or microglia. They proposed that ApoJ deficiency led to a lack of cholesterol supply to retinal neurons for 24S-OHC production by CYP46A1. 24S-OHC being an allosteric activator of NMDA receptors, decreased levels would result in the observed impairment of neuron function. The reduction of retinal cholesterol levels *per se* could also be implicated, regarding the known roles of cholesterol in neuronal membranes (55). A recent review by Pikuleva and Cartier summarizes data regarding the role of CYP46A1 in neurological diseases and presents its potential as a therapeutic target (170).

Altogether, the data detailed above indicate that CYP46A1 expression could be beneficial in the context of glaucoma. As a pathway of cholesterol elimination, it prevents its accumulation in neurons and subsequently its toxicity. Moreover, the CYP46A1 product 24S-OHC appears to have neuroprotective effects. However, evidence indicates that it could also be deleterious. First, the role of 24S-OHC toward Aβ peptide is ambiguous. This is relevant to glaucoma since Aβ peptides have been detected in the aqueous humor of glaucomatous patients (159) and shown to co-localize with apoptotic RGCs and to be neurotoxic for these cells in the rat (158). While 24S-OHC has been reported to inhibit Aβ peptide production (169), it was also shown that it potentiates its apoptotic effects in cultured neurons (171, 172). Of note, different oxysterols might have different effects since 27-OHC has been shown to be much less potent than 24S-OHC in diminishing Aβ peptide secretion (169) or even to promote it in a neuroblastoma cell line (173). Moreover, it has been demonstrated that 24S-OHC activates glutamate receptors (NMDA receptors) in hippocampal neurons (174) and slices (13), and promotes exocytosis of synaptic glutamate (175). This could possibly exacerbate excitotoxicity, death of neurons *via* overactivation of glutamate receptors, one of the proposed mechanisms to explain RGC death and glaucoma development (33).

While the data detailed above suggest a role of oxysterols, especially CYP46A1 product, in glaucoma, the characterization of the retina of mice deficient in CYP enzymes does not absolutely confirm this idea. Instead, these mouse models (*Cyp46a1* KO, *Cyp27a1* KO, and double KO) rather develop a retinal microangiopathy phenotype sharing some similarities to AMD or diabetic retinopathy (60–62). Retinal vascular abnormalities including retinal-choroïdal anastomosis, increased vascular permeability, tortuosity and capillary degeneration were observed. Retinal cholesterol levels were increased, despite normal plasma levels, with deposits at the basal membrane of the RPE as well as in abnormal vessel walls. In the double KO mouse line only, the amplitude of the ERG signal was diminished from 6 months of age indicating altered retinal function. Every model exhibited Müller cell activation. Very recently, however, the same research group conducted a more glaucoma-oriented retinal characterization of *Cyp46a1* KO mice and reported increased IOP and cup-to-disk ratio as well as decreased amplitudes of the pattern ERG (designed for investigation of RGC response), thus pointing to a glaucoma-like phenotype (55). The phenotype of these models is summarized in **Table 1**.

The consequences of hypercholesterolemia were investigated in rats using a cholesterol-supplemented diet for 24 weeks. Their retina exhibited a reduction of cell number in the outer nuclear and ganglion cell layers, swollen inner plexiform and ganglion cell layers as well as increased thickness of the retinal nerve fiber layer. This was associated with an increased expression of nitric oxide synthase (NOS-2) in these areas. Since NOS-2 has been detected in glaucomatous optic nerve heads and NOS-2 inhibition protects against RGC death in a rat model of glaucoma, the authors suggest that excess circulating cholesterol could participate in the disease development by inducing NOS-2 expression and consecutively elevating oxidant tissue injury. Unfortunately, retinal cholesterol levels were not documented in this study (176).

In an experimental model of glaucoma in the rat, we described an alteration of the retinal cholesterol homeostasis (39). Within the first 18 hours following laser-induced intraocular hypertension, genes involved in cholesterol biosynthesis and uptake (*Hmgcr*, *Ldlr*) were upregulated while genes involved in efflux (*ApoE* and *Cyp27a1*) were downregulated. Associated with these hypercholesterogenic changes, retinal levels of cholesterol and its precursors (desmosterol and lathosterol) were elevated 3 days post-laser. Concomitantly, gliosis, inflammation and RGC death were observed. Interestingly, one month later, the retinal cholesterol homeostasis was restored following the activation of hypocholesterogenic mechanisms (upregulation of *Abca1*, *ApoE*, *Cyp27a1* genes and downregulation of *Hmgcr*, *Ldlr* genes). No apoptotic RGC could be detected at this time point. While a causal link is hard to demonstrate, it is tempting to consider that restoration of cholesterol homeostasis was beneficial to RGC viability, considering the known neurotoxic effect of cholesterol. Overall, this study revealed that ocular hypertension, the main risk factor for glaucoma, is associated with transcriptional regulation of the major actors of cholesterol metabolism and transient alterations in sterol levels in the rat retina. It remains difficult to determine whether the perturbations of cholesterol metabolism are a cause or a consequence, or both, of the cellular alterations that are characteristic of glaucoma development, i.e. Müller cell gliosis and RGC death.

A recent study provided another evidence for the possible role of dysregulation of cholesterol homeostasis in glaucoma via the characterization of *Abca1* KO mice (66). The authors reported accumulation of cholesterol in the retina and loss of RGCs as the mice aged, reaching 26% by the age of 1 year. This was associated with altered mitochondrial function and autophagy flux that may lead to RGC degeneration. The role of excess cholesterol in RGC death was further supported by the fact that it was reversed by administration of atorvastatin, an inhibitor of endogenous cholesterol biosynthesis (see **Table 1**). Similar observations had been made previously by Shinozaki et al. (65).

While there is evidence that perturbations of cholesterol metabolism and oxysterols are implicated in glaucoma pathogenesis, the underlying mechanisms and contributions of specific cell types still have to be determined.

## 4 Glia, sterols and glaucoma

As developed in the present review, Müller cells are major contributors of retinal cholesterol metabolism. Through a cooperation with neurons, they participate in maintaining cholesterol homeostasis in the retina (see section 2). Any dysregulation of cholesterol metabolism in Müller cells could therefore affect RGCs and glaucoma development. It is well known that Müller cells undergo activation during the course of glaucoma and some evidence indicate that cholesterol homeostasis might be perturbated (see section 3). This prompts the idea of an association between these two events even though concomitancy is not sufficient to prove a causal link. This section gives an overview of data-based hypotheses for the implication of retinal cholesterol metabolism in Müller cell response to glaucomatous injury, and *vice versa*.

The most abundant data in that context relate to CYP46A1 and its product 24S-OHC. As mentioned above (section 3.2), experimental models of glaucoma are associated with early increases in retinal CYP46A1 expression and 24S-OHC levels. It was reported both in an *ex vivo* retina 24h after an exposure to high pressure (9) as well as *in vivo* in a rat model of laser-induced elevated intraocular pressure from 3 days post-intervention (36). 24S-OHC being produced from cholesterol via the action of CYP46A1, expressed by RGCs, this could be a consequence of cholesterol release due to RGC suffering and apoptotic death, which was observed in these models (9, 39). This idea has already been proposed for early stages of degenerative diseases of the CNS. Increased 24S-OHC levels in the cerebrospinal fluid and plasma of AD patients at early stages is indeed thought to result from increased brain cholesterol turnover and neuron cell degradation (11). In the context of the neuron-glia communication described earlier (section 2.2.4), elevated 24S-OHC levels are likely to modulate cholesterol metabolism in Müller cells, as it was shown *in vitro* in primary cultures (92). Indeed, 24S-OHC exposure induced a decrease in cholesterol biosynthesis as well as activation of cholesterol efflux pathways, both *via* LPs and CYP27A1 (**Figure 4**). These regulatory mechanisms would thus contribute to avoiding cholesterol overload in the retina, which would be deleterious, especially for the weakened RGCs. Such hypocholesterogenic mechanisms – namely decreased cholesterol biosynthesis and uptake, increased cholesterol efflux - have actually been shown in the retina *in vivo* in a rat model of glaucoma from 3 days post experimental elevation of IOP even though surprisingly no change in 24S-OHC levels could be measured (39). Especially, the gene expression of *Hmgcr* and *Cyp27a1*, which are more specific of glial cells, were decreased and increased, respectively, supporting an implication of Müller cells.

At late time-points (from 30 days post-laser), in the previously mentioned-study by Fourgeux et al., retinal CYP46A1 levels were maintained, and even slightly increased in an experimental glaucoma model in the rat, despite the expected loss of CYP46A1-expressing RGCs characteristic of this model (36). Since glial activation was strong at these time-points, CYP46A1 expression might be due to glial expression of this otherwise RGC-specific enzyme in order to maintain retinal CYP46A1 levels, which are crucial for cholesterol homeostasis. Again, this hypothesis had already been made in the brain in the context of AD. Authors reported that CYP46A1 expression was decreased in neurons but increased in astrocytes in the brain of AD patients (168, 169). They suggested that glial compensation for the loss of CYP46A1 neuronal expression may be of some benefit for the patients by decreasing cholesterol concentration in the brain, since it has been shown that diminished brain cholesterol levels may decrease the tendency of Aβ peptide to aggregate. Alike gliosis in glaucoma, which is dynamic and presents an ambivalent role, the modifications of cholesterol metabolism in Müller cells during glaucoma could be both beneficial and detrimental to RGCs. At some point, it would be possible for the overexpression of CYP46A1 to become deleterious. Indeed, an overexpression of CYP46A1 inducing a reduction of membrane cholesterol has been shown to reduce glutamate uptake *via* the transporter EAAT2 in astrocytes, which are in charge of glutamate clearance (177). This function is ensured by Müller cells in the retina and is known to prevent excitotoxicity, a pathological mechanism contributing to glaucoma (33). Moreover, 24S-OHC can activate glutamate receptors (NMDA receptors) in neurons (13, 174), and therefore promote excitotoxicity under specific stressful conditions.

Recently, the implication of glial cholesterol metabolism, especially *via* ABCA1, in glaucoma, has been elegantly demonstrated. Using a glia-specific *Abca1*-KO mouse model (*Abca1* ^flox/flox^;GFAP-Cre), Shinozaki et al. showed that ABCA1 deficiency in glial cells causes a optic neuropathy-like phenotype targeting RGCs (65). Although ABCA1 ablation in this model should also affect Müller cells, the authors considered that the phenotype was due to ABCA1 deficiency in astrocytes since they observed that ABCA1 was mainly expressed in this glial cell type. Results showed RGC degeneration, regional loss of axons in the optic nerve and retinal dysfunction in aged mice, without change in IOP. Accumulation of cholesterol in the nuclear fiber layer where astrocytes are present was observed. By contrast, cholesterol content was lower in the GCL and aqueous humor. Inflammation was also detected (**Table 1**). Overall, the authors proposed that an intracellular accumulation of cholesterol in astrocytes might over time induce inflammation, which could sensitize the surrounding RGCs to excitotoxicity. This could be exacerbated by astrocytic dysfunction since it was also shown that cultured KO astrocytes exhibited increased extracellular glutamate levels suggesting that ABCA1 deficiency may enhance release or reduced uptake of the neurotransmitter by astrocytes. Besides, the authors proposed that an inadequate supply of cholesterol to RGCs might be implicated in the observed neurodegeneration since glia-derived cholesterol is thought to be required for synaptogenesis of RGCs. This study thus strongly suggests the importance of RGCs-astrocytes interactions and cholesterol metabolism in the context of glaucoma.

## 5 Concluding remarks and future directions

Neuron-glia interaction and cooperation are crucial for the maintenance of homeostasis and proper functioning of the CNS, including the retina. Evidence presented in the present review supports the fact that cholesterol metabolism relies on these interactions in the retina, especially between Müller cells and RGCs, and that it might be relevant to glaucoma pathophysiology. Deciphering neuron-glia interactions and the specific contribution of each cell type in the retina, in health and disease states, requires to determine what is happening specifically in the different cell types. This remains quite challenging *in vivo* and cell type-specific (Müller cells, astrocytes, microglia, RGCs) experiments would be needed. Rodent models with cell-specific knock-out of genes involved in cholesterol metabolism or with overexpression of proteins of cholesterol metabolism under the control of cell-specific drivers or mediated by viruses with cell-specific tropism would be useful tools to develop. Immunofluorescence, especially colocalization experiments, and *in situ* hybridization techniques can also provide some information. Moreover, the current boom of MALDI-MS (matrix-assisted laser desorption ionization-mass spectrometry), which consists in mass spectrometry analysis of molecules directly on histological tissue sections, will inevitably offer great analytic perspectives for retinal studies. Single-cell RNA sequencing can also represent a powerful tool to analyze gene expression profiles, especially for cell types accounting for a small proportion of retinal cells, such as RGCs. Finally, *in vitro* and *ex vivo* techniques such as co-culture of different cell types or retinal organoïds to mimic *in vivo* interactions should provide useful pieces of information.

Retinal cholesterol metabolism exhibits the peculiarity to rely both on *in situ* events (biosynthesis, intraretinal transport and exchanges) and systemic cholesterol status (*via* exchanges with the circulation). Another challenge is therefore to better understand the role of cholesterolemia in retinal cholesterol homeostasis. An important point is to determine whether circulating cholesterol, which is accessible to analysis by blood sampling and to dietary and pharmacological interventions, represents and influences retinal cholesterol.

Finally, oxysterols appear as promising targets to prevent or correct dysregulation of cholesterol homeostasis and neurodegenerative diseases. CYP46A1 and 24S-OHC are currently the subjects of many reports focusing on their action in the brain, on cholesterol homeostasis and beyond, and on their potential as therapeutic targets for degenerative diseases of the CNS or neuropsychiatric disorders (14, 170, 178, 179). Other bioactive oxysterols of the retina, such as 27-OHC and 27-COOH, as well as neurosteroïds, such as pregnenolone, which is already a matter of interest for brain diseases (180) and is a product of the CYP11A1 enzyme also expressed in the retina, will deserve further investigations in the future.

## Author contributions

EM: Writing – original draft, Writing – review & editing. JS: Writing – original draft, Writing – review & editing. EL: Writing – original draft, Writing – review & editing. NA: Writing – review & editing.
